# Habenula–ventral tegmental area functional coupling and risk aversion in humans

**DOI:** 10.1073/pnas.2500815122

**Published:** 2025-10-30

**Authors:** Wanjun Lin, Jiahua Xu, Xiaoying Zhang, Raymond J. Dolan

**Affiliations:** ^a^Max Planck University College London Centre for Computational Psychiatry and Ageing Research, University College London, Queen Square Institute of Neurology, London WC1B 5EH, United Kingdom; ^b^Department of Clinical Medicine, Center for Functionally Integrative Neuroscience, Aarhus University, Aarhus C 8000, Denmark; ^c^Psychiatry Research Center, Beijing Huilongguan Hospital, Peking University Huilonguan Clinical Medical School, Beijing 100096, China; ^d^State Key Laboratory of Cognitive Neuroscience and Learning, International Data Group/McGovern Institute for Brain Research, Beijing Normal University, Beijing 100875, China; ^e^Wellcome Centre for Human Neuroimaging, University College London, Queen Square Institute of Neurology, London WC1N 3AR, United Kingdom

**Keywords:** habenula, ventral tegmental area, uncertianty, reinforcement learning, computational modeling

## Abstract

Maladaptive responses to uncertainty, including excessive risk seeking or avoidance, are linked to a range of mental disorders. One expression of these is a provariance bias (PVB), i.e., risk-seeking manifests as a preference for choosing options with higher variances/uncertainty. Using a magnitude learning task, we show that individual differences in PVB are explained by a model that includes asymmetric learning rates, allowing differential learning from positive prediction errors (PPEs) and negative prediction errors (NPEs). Using high-resolution 7T functional MRI (fMRI), we identify distinct neural responses to PPEs and NPEs in value-sensitive regions, including habenula (Hb), ventral tegmental area (VTA), nucleus accumbens (NAcc), and ventral medial prefrontal cortex (vmPFC) in humans. Notably, prediction error signals in NAcc and vmPFC were boosted for high variance options. NPEs responses in NAcc were associated with a negative bias in learning rates that was linked to the strength of Hb-VTA negative functional coupling during NPE encoding, with a mediation analysis revealing this coupling influenced NAcc responses to NPEs via an impact on learning rate bias. Our findings implicate Hb-VTA functional coupling in the emergence of risk preferences during learning, with implications for psychopathology.

## Significance

Dealing with uncertainty is crucial for survival where significant individual differences exist. In general, people show a preference for options with higher variance (uncertainty), known as a provariance bias (PVB). Using a bespoke learning task, we show that individual differences in PVB reflect differential learning from good and bad outcomes. Neurally, using ultra-high-resolution neuroimaging, functional interactions between habenula (Hb) and ventral tegmental area (VTA) are associated with this learning bias. Specifically, a greater negative learning bias correlates with an enhanced Hb–VTA negative functional connectivity during worse-than-expected outcomes. The findings highlight the importance of functional coupling between regions encoding punishment (Hb) and reward (VTA) in risk-sensitive behaviour, with implications for understanding maladaptive responses to uncertainty associated with many mental problems.

Decision-making under uncertainty is a daily challenge, with maladaptive responses proposed as a mechanistic account for a range of psychopathologies, including anxiety (1, 2), depression (3, 4) and gambling disorders (5). More specifically, risk aversion manifesting as a disposition to prefer an option with lower uncertainty/variance (6) is linked to higher anxiety and depression scores in the general population (7). This renders it important to understand how risk-related biases emerge as individuals deal with an uncertain environment.

**Fig. 1.**
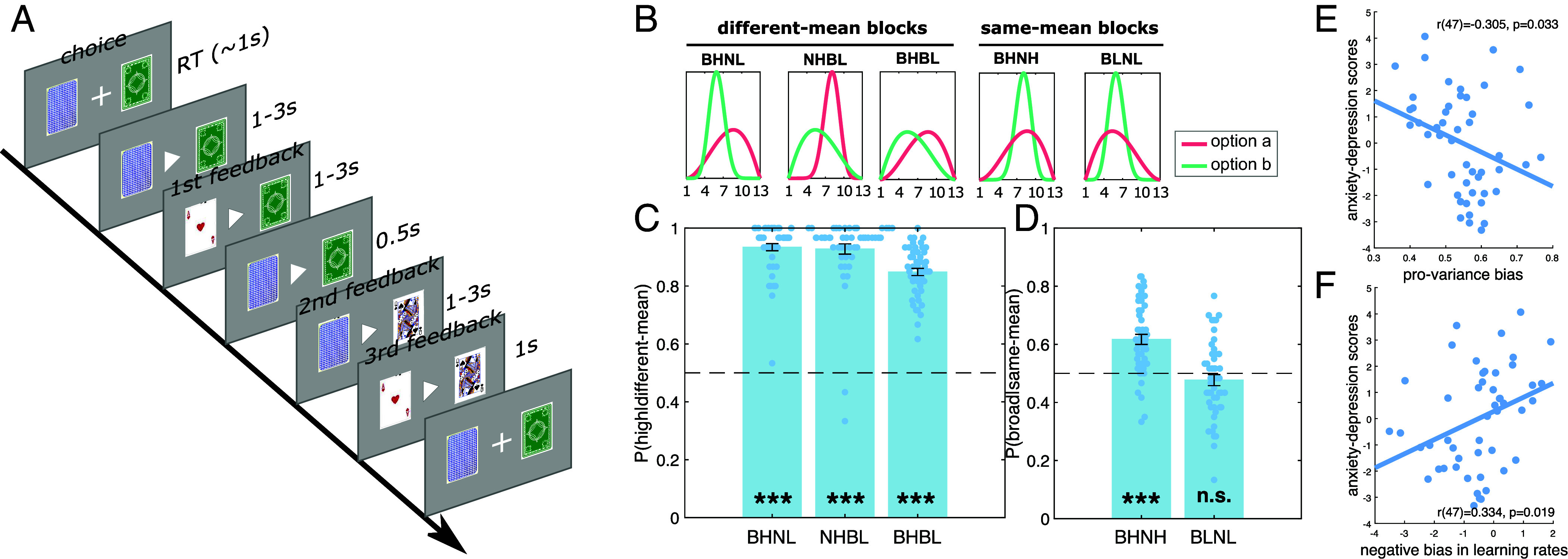
Experimental design. (*A*) Task structure. Within a task block, participants were presented with the same two card decks across 30 trials and asked to choose from one of the decks (choice). After entering a choice, a triangle in the center of the screen pointed to the chosen deck. Following this, a card from each deck was shown sequentially to participants (1st feedback and 2nd feedback, 1 to 3 s), with the order of presentation counterbalanced across trials, with the two cards then shown together for 0.5 s (3rd feedback). (*B*) Block types. The two card decks in a block could have the same or different means (high or low), and the same or different variances (narrow or broad). This entailed eight blocks in all comprising four different-mean blocks [one broad-high vs. narrow-low (BHNL) block, one narrow-high vs. broad-low (NHBL), and two broad-high vs. broad-low (BHBL) blocks] and four same-mean blocks [two broad-high vs. narrow-high (BHNH) and two broad-low vs. narrow-low (BLNL)]. The block order was counterbalanced across participants (see *Methods* for details). (*C*) The percentage of choosing the higher option for the different-mean blocks, with dots (light blue) representing data points for each participant for that block type. (*D*) The percentage of choosing the broader option for the same-mean blocks. The dots represent data points for each participant for that block type. (*E*) PVB negatively correlated with anxiety-depression scores. (*F*) Model-derived negative bias in learning rates correlated with the anxiety-depression scores. Error bars indicate SE; *P < 0.05; **P < 0.01; ***P < 0.001.

We developed a simple magnitude learning task (8), wherein participants learn and choose between options with different value distributions (varying means and/or variances, also see Fig. 1*B*). This task enables manipulation of distribution variance/uncertainty independently from its mean, in contrast to probabilistic learning tasks where risk uncertainty and value are related (9). Previous studies show that when presented with two options, drawn from value distributions with the same mean but different variances, human and nonhuman primates show a preference for an option with the higher variance, the very opposite to risk aversion, and known as “provariance bias” (PVB) (10, 11).

Previously we showed, both in simulations and empirical data, that a modified Rescorla–Wagner (RW) learning model (12), i.e., a two-learning-rate RW model (2lr-RW), captured individual differences in a PVB (8). In brief, this 2lr-RW model incorporated separate learning rates (LR) for positive (PPEs) and negative (NPEs) reward prediction errors [a better or worse outcome relative to expected values (EVs) respectively], allowing EVs to differ from the true outcome means. Thus, a positive-LR-biased agent, with a higher positive compared to negative learning rate, will integrate more PPEs than NPEs into their EVs compared to NPEs during learning, leading to enhanced EVs in a higher variance environment and the emergence of a PVB, i.e., risk-seeking. We note evidence from congenital learned helpless (cLH) rats, who manifest a negative learning bias, also show less risky choices (13). In the current study, we ask which brain regions are associated with such learning rate bias that could give rise to individual differences in risk preferences.

Dopamine (DA) in particular is linked to risk behavior as evidenced by an impact of dopaminergic medication on risk-related decision-making (14–16). Optogenetic activation of the midbrain DA neurons has provided evidence for a causal influence on risky decisions (17, 18). DA neurons in the ventral tegmental area (VTA) have a central role in motivation and learning (19, 20) by signaling reward prediction errors (RPEs) via increased activity for unexpected rewards and decreased activity for unexpected reward omissions (21, 22). More recently, the lateral habenula (Hb) has been shown to negatively mirror DA RPEs (23–25). Moreover, stimulation of Hb neurons inhibits midbrain DA neurons (23), suggesting lateral Hb is a source of NPE signaling to DA neurons. Critically, lesioning the lateral Hb attenuates an inhibition of the VTA DA neuron activity seen in response to reward omissions (26), resulting in a decrease in negative learning rates relative to positive learning rates (i.e., a positive learning bias) (26). On this basis, we specifically ask whether BOLD responses to PPEs and NPEs in human VTA and Hb, and their functional connectivity, relate to a learning rate bias estimated using our magnitude learning task.

Animal literature shows that VTA DAergic neurons have strong projections to nucleus accumbens (NAcc) and medial prefrontal cortex (mPFC) (27, 28), with both regions expressing robust RPEs signals (29, 30). At a behavioral level, phasic stimulation of NAcc dopamine receptor type-2 (D2R)-expressing cells increases risk aversion, while pharmacological inactivation of mPFC increases risky choices (31, 32). On this basis, we were interested in examining whether activity in downstream dopaminergic regions (NAcc and mPFC) are associated with individual differences in risk-preferences (PVB) in humans. Additionally, a recent study indicates that NAcc encodes variance in outcomes, consistent with a suggestion that NAcc involved in a representation of reward distribution (33). Similarly, it has been shown that mPFC computes state uncertainty (34) as well as encoding a reward distribution (35).

In the current study, we investigate the neural mechanisms underlying individual differences in PVB. Using a bespoke magnitude learning task (8), combined with partial field-of-view (FOV) ultra-high field (7T) fMRI, we examined whether a priori regions of interest (ROIs) that included Hb, VTA, NAcc, and ventral medial prefrontal cortex (vmPFC) encode trial-by-trial PPEs and NPEs. Critically, we also asked whether functional coupling between regions that are associated with task-related value-updating relates to individual differences in PVB.

## Results

### Lower Provariance Bias Is Associated with Higher Anxiety and Depression.

Fifty-seven participants recruited from the general population performed a magnitude learning task (Fig. 1*A*) while inside a 7T scanner [eight were excluded from analyses based on prespecified criteria (see *Methods* for details)]. To introduce variation in PVB, we recruited participants with a spectrum of anxiety and depression scores, features previously linked to individual differences in risky decision-making (7). Participants chose between two card decks associated with different value distributions, wherein we independently manipulated the variance and mean of the distributions (Fig. 1*B*). Critically, in half of the blocks (4/8), participants were presented with options with identical means but different variances (the same-mean blocks; see Fig. 1*B*), enabling indexing of how choices were impacted by variance.

We found that, for different-mean blocks, the choice percentage for the higher mean option was significantly above chance level (0.5) (see Fig. 1 *B* and *C*, all t(48) > 24.111, *P* < 0.001). As expected, participants performed less well in the BHBL (both options with high variance) blocks compared to blocks where one of the distributions was narrow (i.e., the NHBL and BHNL block) (both t(48) < −4.020, *P* < 0.001). These data indicate participants learned the task, with higher uncertainty impacting learning a value difference.

We next examined whether, for blocks with the same mean, there was a preference for the broader options. Consistent with previous findings (8), participants on average preferred the broader over the narrower option in the both-high (BHNH) blocks (t(48) = 6.717, *P* < 0.001). However, for the both-low (BLNL) blocks, the percentage of choosing the broader option did not differ from chance level (t(48) = −1.215, *P* = 0.230). These results suggest participants in general manifest a provariance bias, particularly for the both-high blocks. These results were confirmed using a mixed effects generalized linear model (GLM), which revealed a significant main effect of mean (beta coefficient = 1.817, 95% CI [1.712, 1.922], *P* < 0.001) and a mean*variance on participants’ choices (beta coefficient = 0.367, 95% CI [0.253, 0.481], *P* < 0.001; see *SI Appendix*, *Methods*).

Next, we asked whether individual differences in provariance bias (mean over all the same-mean blocks) were associated with task-independent measures of individual differences in self-reported anxiety and depression. We found a provariance bias was negatively correlated with State-Trait Anxiety Inventory questionnaire scores (STAI) (r(47) = −0.314, *P* = 0.028) and marginally with the Zung self-rating depression scale (SDS) (r(47) = −0.280, *P* = 0.051). These results replicated findings from an independent online pilot study (n = 213): r(211) = 0.174, *P* = 0.011 for the STAI, and r(211) = −0.185, *P* = 0.007 for the SDS (*SI Appendix*, Fig. S1 and *Methods*). To simplify the presentation, we provide overall scores for anxiety and depression (Anxiety-Depression scores) by summing the z scores of the SDS and STAI; see Fig. 1*E*, r(47) = −0.305, *P* = 0.033). Again, these results were confirmed by mixed effect GLM, which showed a significant three-way interaction of mean*variance*anxiety-depression scores (beta coefficient = −0.031, 95% CI [−0.051, −0.009], *P* = 0.006).

Provariance bias could arise from a lack of symmetry in integrating positive and negative prediction errors (NPEs) into prior expectations. Using computational modeling we found that, compared to a suite of alternatives, a 2lr-RW was the best-fitting model (*SI Appendix*, Fig. S2*A*; see details in *Methods* and *SI Appendix*). Consistent with a hypothesis of a learning bias, model-derived negative biases in learning rates (the ratio of the negative learning rate to the sum of both learning rates; see *Methods*) accounted for individual differences in PVB (*SI Appendix*, Fig. S3, r(47) = −0.840, *P* < 0.001), and correlated with Anxiety-Depression scores (r(47) = −0.334, *P* = 0.019, Fig. 1*F*). This suggests that negatively biased learning is a potential computational mechanism underlying an attenuation of a provariance bias (or increased risk avoidance), particularly in people with higher anxiety and depression trait scores.

### Habenula Encodes NPEs in Humans.

**Fig. 2.**
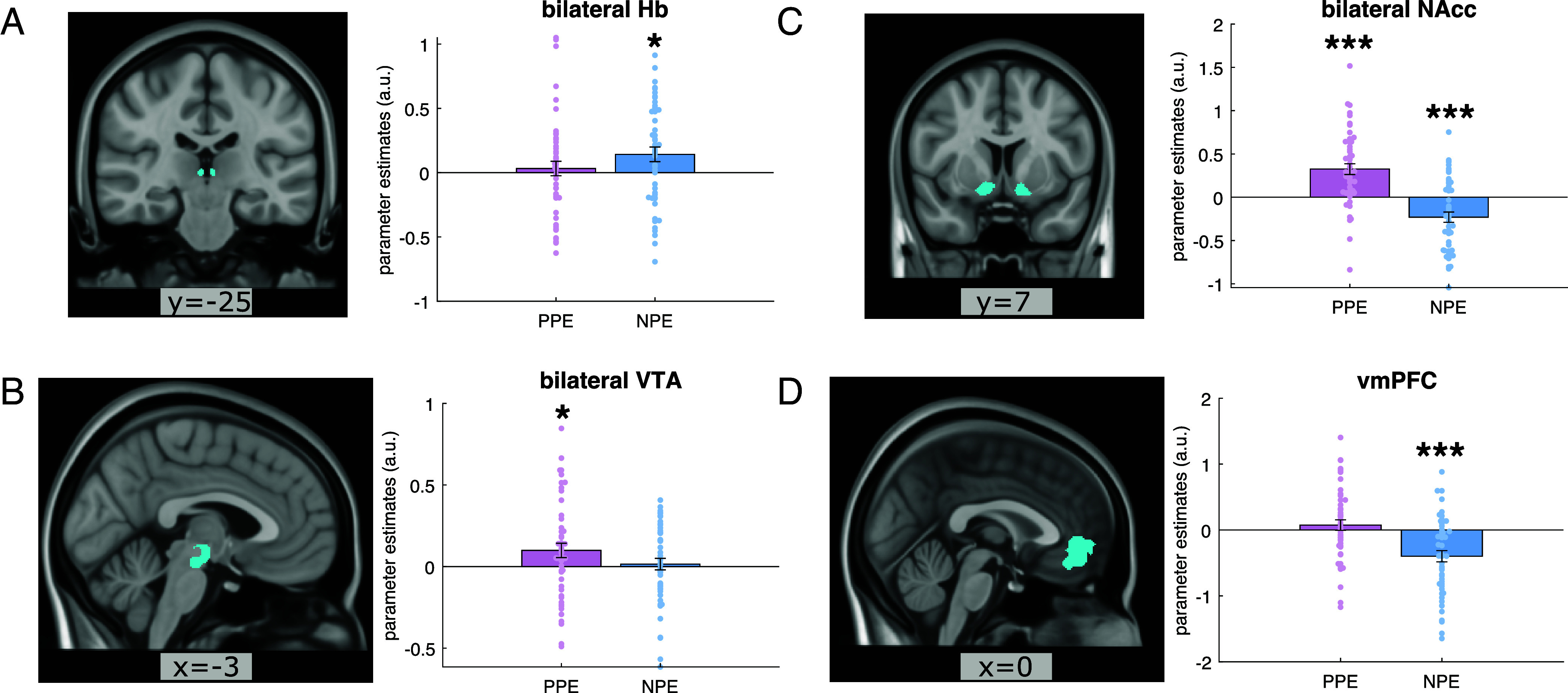
Regions encode prediction errors. (*A*) BOLD responses in the bilateral habenula (Hb) were positively modulated by NPEs (*P* = 0.016), but not by positive prediction errors (PPEs) (*P* = 0.571). *Left*: a coronal view of the bilateral Hb anatomical mask. *Right*: parameter estimates of BOLD responses to PPE and NPE, respectively, within bilateral Hb anatomical mask. (*B*) BOLD responses in the bilateral VTA showing positive modulation to PPEs (*P* = 0.028) but not to NPEs (*P* = 0.672). *Left*: a sagittal view of VTA anatomical mask. *Right*: parameter estimates of BOLD responses to PPE and NPE, respectively, in this bilateral VTA anatomical mask. (*C*) BOLD responses in bilateral nucleus accumbens (NAcc) were positively modulated by PPE and negatively by NPE (both *P* < 0.001). *Left*: a coronal view of the bilateral NAcc anatomical mask. *Right*: parameter estimates of BOLD responses to PPE and NPE in this bilateral NAcc anatomical mask. (*D*) BOLD responses in the vmPFC were negatively modulated by NPE (*P* < 0.001) but not PPE (*P* = 0.348). *Left*: a sagittal view of the vmPFC functional defined mask. Right parameter estimates of BOLD responses to PPE and NPE, respectively, in this vmPFC mask. Each dot on the bar graph represents a parameter estimate for each participant. Error bars indicate SE; *P < 0.05; **P < 0.01; ***P < 0.001.

We next examined a candidate neural basis for the observed PVB by first ascertaining the expression of PPEs and NPEs within a priori ROIs, namely the Hb, VTA, NAcc, and vmPFC. Here, we used anatomical masks (28) for NAcc and VTA, and a functionally defined ROI for vmPFC, which was derived from a meta-analysis of value-based decision-making tasks (Fig. 2 *B*–*D*) (36). Due to the relatively small size of the Hb (around 30 mm^3^ in volume in humans) (37), and to derive a more precise study-based Hb ROI based on the subjects’ ultrahigh-resolution T1 images (0.7 mm^3^), we manually drew an anatomical Hb mask in each participant for each hemisphere following previous literature (37–39) (see *Methods* for more details). We ran a model-based fMRI analysis with trial-by-trial PPEs and NPEs for the chosen option calculated using the best-fitted parameters for each participant from the 2lr-RW model (*Computational Modeling and GLM 1*).

At choice outcome, VTA BOLD responses were positively modulated by trial-by-trial PPEs (t(48) = 2.261, *P*_uncorrected_ = 0.028, *P*_false recovery rate (FDR)_ = 0.045; see Fig. 2*B*), but not by NPEs (*P*_uncorrected_ = 0.672). To examine how well our data suggested that VTA BOLD responses were not modulated by NPEs, we ran a Bayesian one-sample *t* test, an approach that can be used to determine whether nonsignificant results support a null hypothesis (40), with the alternative hypothesis (H1) that the beta estimates for NPEs regressors are different from zero. The results indicated moderate evidence that supported the null hypothesis (H0, i.e., not different from zero), with Bayesian Factor (BF_10_) = 0.169, posterior median δ = 0.057, 95% CI [−0.213, 0.328], under the default Cauchy prior r = 0.707. We also tested the BF supporting VTA BOLD responses were positively modulated by PPEs (with the alternative hypothesis H+ that the beta estimates for NPEs regressors is greater than zero). The results suggested moderate evidence for the H+ (BF_+0_ = 3.09, δ = 0.307, CI [0.056, 0.587]; see *SI Appendix*, Fig. S1 for the BF Robustness check plots for all the ROIs).

NAcc BOLD responses were modulated by both PPEs and NPEs (Fig. 2*C*), involving enhanced activation to PPEs (t(48) = 5.028, *P*_uncorrected_ < 0.001, *P*_FDR_ < 0.001) and a stronger deactivation to higher NPEs (t(48) = −3.886, *P*_uncorrected_ < 0.001, *P*_FDR_ < 0.001). The vmPFC also showed a relative deactivation as a function of NPEs (t(48) = −4.651, *P*_uncorrected_ < 0.001, *P*_FDR_ < 0.001, Fig. 2*D*) but no significant modulation by PPEs (*P*_uncorrected_ = 0.348, BF_10_ = 0.237 (moderate evidence for H0), δ = 0.127, CI [−0.145, 0.400]). Whole-FOV (partial FOV data) group-level results for the NPEs and PPEs accorded with these ROI results (*SI Appendix*, Fig. S5).

In bilateral Hb, combining left and right Hb masks, we found a positive response scaled by NPEs (see Fig. 2*A*, t(48) = 2.494, *P*_uncorrected_ = 0.016, *P*_FDR_ = 0.032, BF_+0_ = 4.992 (moderate evidence for H+), δ = 0.337, CI [0.075, 0.621]) but not to PPEs (*P*_uncorrected_ = 0.571, BF_10_ = 0.181 (moderate evidence for H0), δ = 0.076, CI [−0.194, 0.348]). A broadly similar pattern of results was evident when we examined the left (NPE: t(48) = 1.965, *P*_uncorrected_ = 0.055) and right Hb (NPE: t(48) = 2.223, *P*_uncorrected_ = 0.031) alone (*SI Appendix*, Fig. S6). The overall pattern of results is consistent with habenula encoding NPEs, where worse-than-expected outcomes engender a stronger BOLD signal.

**Fig. 3.**
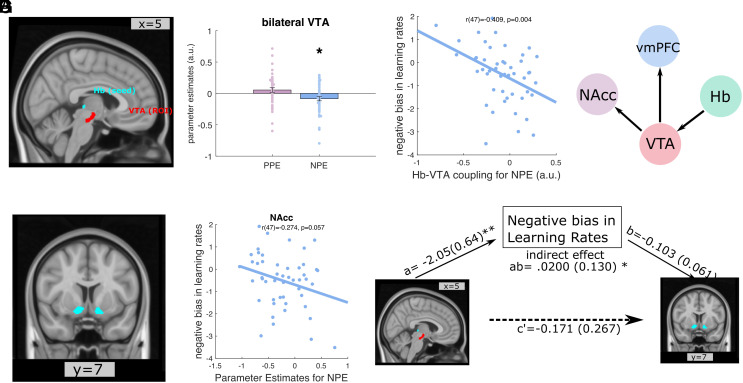
Negative functional coupling during NPEs between Hb and VTA related to negative learning rate bias and NAcc BOLD response to NPEs. (*A*) A coronal view of the Hb (PPI seed) and VTA (ROI) anatomical mask. (*B*) A bar graph depicting parameter estimates of Hb-VTA functional connectivity strength as a function of PPEs and NPEs, respectively. Each dot on the bar graph represents a parameter estimate for each participant. Error bars indicate SE. *P < 0.05. (*C*) A scatter plot for the correlation between the Hb-VTA functional connectivity during NPEs encoding (x-axis) and negative bias in learning rates (y-axis) (*P* = 0.004). (*D*) The winning model of Structural Equation Modeling analysis. (*E*) A coronal view of the bilateral NAcc anatomical mask. (*F*) A scatter plot for the correlation between the parameter estimates of BOLD responses in the bilateral NAcc to NPE (x-axis) and negative bias in learning rates (y-axis). (*G*) Mediation effect of learning rates bias. Hb-VTA coupling influenced the NAcc BOLD responses to NPEs through the mediation of the learning bias (mediation *P* = 0.0367). Hb: Habenula; VTA: ventral tegmental area; NAcc: nucleus accumbens; vmPFC: ventral medial prefrontal cortex; PPEs: positive reward prediction errors. NPEs: negative reward prediction errors.

We next asked whether BOLD responses to PPEs and NPEs in target ROIs (i.e., VTA, NAcc, Hb, and vmPFC) related to a bias in learning rates. We found a marginally significant negative association between NAcc responses to NPEs and a negative bias in learning rates (see Fig. 3*B*, r(47) = −0.274, *P*_uncorrected_ = 0.057). While this suggests NAcc encoding of NPEs relates to a greater negative learning bias, we caution that the effect is not strong and below a conventional significance level.

### Habenula–VTA Functional Connectivity and Learning Rates.

Rodent evidence indicates that inactivating lateral Hb induces a positive learning rate bias, mediated via attenuation of an inhibitory response within VTA to reward omission, i.e., a negative reward prediction error (26). On this basis, we hypothesized that functional connectivity between Hb and VTA would relate to a learning rate bias. To test this, we ran a psychophysiological interaction (PPI) analysis, with bilateral Hb as the seed region (*Methods*). This revealed a significant negative functional connectivity between Hb (seed) and bilateral VTA (ROI) during NPEs encoding (t(48)= −2.479, *P*_uncorrected_ = 0.017, *P*_FDR_ = 0.034, BF_−0_ = 4.835 (moderate evidence for H−), δ = −0.335, CI [−0.619, -0.073]; see Fig. 3*B*), consistent with evidence from animal studies (23, 26), but not for PPEs (t(48) = 1.540, *P*_uncorrected_ = 0.130). Thus, the worse an outcome relative to expectation, the greater the negative coupling between Hb and VTA. Importantly, as predicted, we found a greater negative habenula–VTA connectivity during NPEs encoding related to a more negative bias in learning rates (Fig. 3*C*, r(47) = −0.409, *P*_uncorrected_ = 0.004, *P*_FDR_ = 0.008).

Rodent evidence suggested that Hb inhibits VTA (41, 42) activities to reward omission, consistent with our PPI results that showed a greater negative coupling between Hb and VTA in response to larger NPEs. However, PPI analysis could not indicate the influence relationship between the ROIs. To formally test this proposed functional network, we used a structural equation model (SEM), a tool for analyzing functional and effective connectivity (43, 44), to probe directionality of influence in connectivity between our a priori ROIs (Hb, VTA, NAcc, and vmPFC) (43, 45) (*Methods*). We derived a model of the interaction between these ROIs based on animal studies (for a review see ref. 46), with the Hb influencing VTA first, and NAcc and mPFC then both being subject to an influence from VTA (Fig. 3*D*). The path coefficients from this model were all significantly different from zero (*SI Appendix*, Table S1). We also tested this model against alternative models of similar complexity (see *SI Appendix*, Fig. S8*B* for all the models). The proposed model was the best-fitted model indicated by Akaike Information Criterion (AIC). The model evidence (AICs) from SEM suggested that an interaction between ROI regions was more likely to originate from Hb than from any of the other three ROIs (all three models starting from Hb had lower AICs than models starting with the other ROIs; see *SI Appendix*, Fig. S8*A*).

So far, our results suggested that the interaction between our a priori ROIs is consistent with animal evidence, that Hb influences VTA which then influences NAcc. We showed above that Hb-VTA functional connectivity during NPEs was associated with a negative bias in learning rates, which was also associated with NAcc BOLD activation to NPEs. Here, we probed this interaction in additional mediation analyses (47). Specifically, we asked whether the learning rate bias mediated the NPEs effects on the Hb-VTA functional connectivity and NAcc BOLD responses. This revealed a Hb-VTA functional coupling impacted the NAcc BOLD responses to NPEs through an influence on a learning bias (indirect effect ab = 0.0200 (0.130),95% CI [0.02, 0.56], *P* = 0.0367; see Fig. 3*G*).

### Variance Enhances Prediction Error Signals in the NAcc and the vmPFC.

We hypothesized that a preference for broader options (PVB) emerges because higher variances produce more prediction errors which booted the effects of learning bias generated by an unbalanced positive and negative learning rates (as suggested by our 2lr-RW model). On this basis, we aimed to test whether positive and negative prediction error signals were boosted for the broader compared to the narrower option, i.e., option-specific signed reward prediction errors (so far, we have examined the reward prediction errors for the chosen outcome regardless which option was chosen for a given trial). To do this, we limited the analysis to the equal-mean blocks, where the two options were chosen approximately equally in a given block. Then, we found that we had very few or no trials for a signed prediction error for a specific option type in some participants. Therefore, here we combined PPE and NPE as a single RPE regressor for each option and instead examine whether reward prediction errors BOLD responses in general are larger for a broader than a narrower option. Thus, we ran a new first-level GLM design (*GLM 2*) on fMRI data, where we modeled prediction errors for the broader and narrower options when they were chosen and not-chosen respectively.

**Fig. 4.**
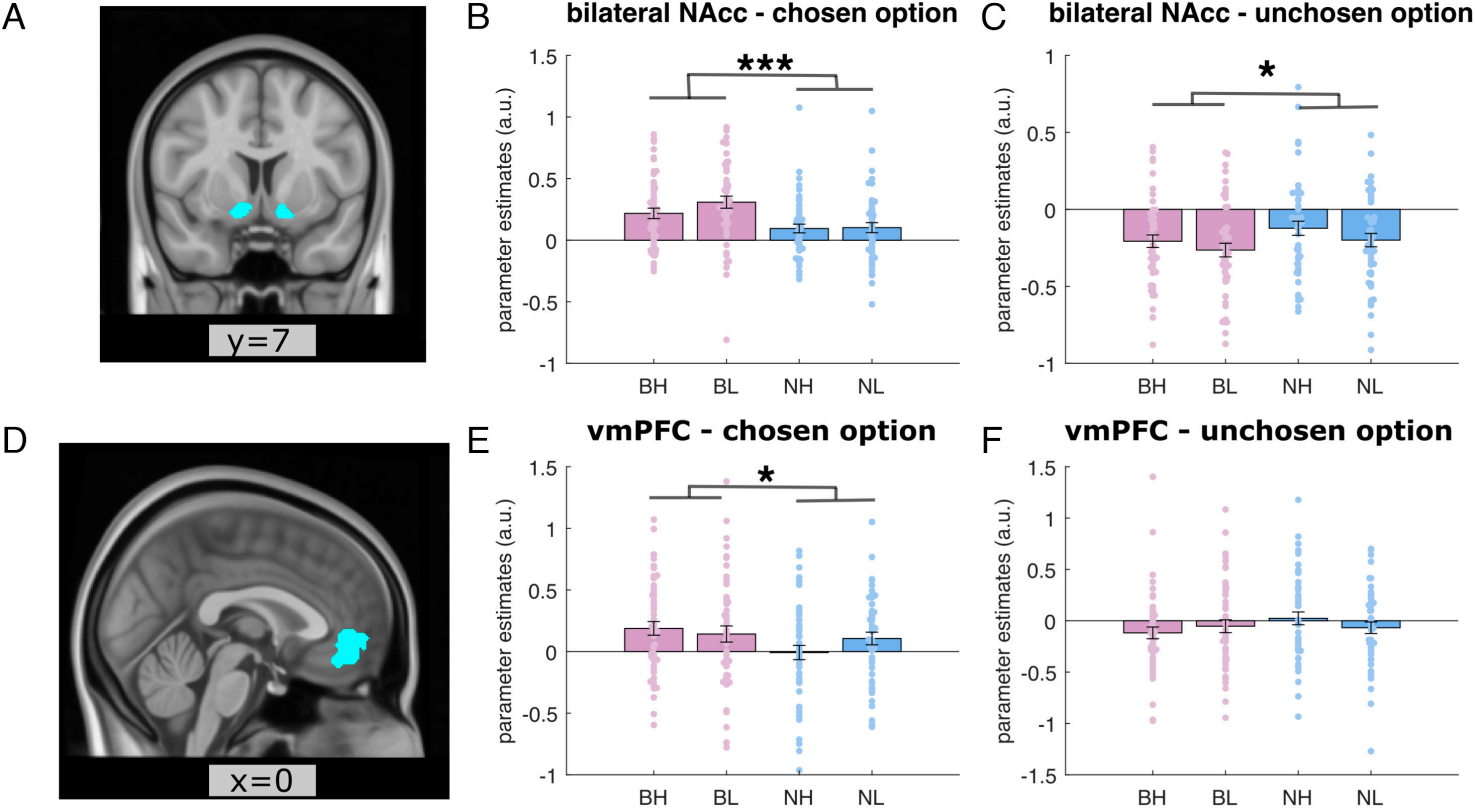
Variance boost prediction error signals in NAcc and vmPFC. (*A*) A coronal view of the bilateral NAcc anatomical mask. (*B* and *C*) Parameter estimates of BOLD responses in this bilateral NAcc anatomical mask to prediction errors for each distribution type for the chosen (*B*) and unchosen option (*C*) respectively in the equal-mean blocks. (*D*) A coronal view of the vmPFC mask. (*E* and *F*) Parameter estimates of BOLD responses in this vmPFC mask to prediction errors for each distribution type for the chosen (*E*) and unchosen option (*F*), respectively, in the equal-mean blocks. BH: broad-high, BL: broad-low, NH: narrow-high, NL: narrow-low. Each dot on the bar graph represents a parameter estimate for each participant. Error bars indicate SE; *P < 0.05; ***P < 0.001.

For prediction error signal for the chosen option, a variance (broad vs. narrow) × mean (high vs. low) repeated ANOVAs indicated a significant main effect of variance in bilateral NAcc (Fig. 4*B*, F(1,48) = 15.298, *P*_uncorrected_ < 0.001) and vmPFC (Fig. 4*E*, F(1,48) = 4.743, *P*_uncorrected_ = 0.034), but not in habenula or VTA (all *P*_uncorrected_ > 0.419). Additionally, we also ran the same analyses for the unchosen outcome which revealed that NAcc encoded prediction errors for the unchosen outcome in the opposite direction, i.e., lower the BOLD response to higher prediction errors (all t < –2.665, *P*_uncorrected_ < 0.010), where repeated ANOVAs also revealed a significant main effect of variance in bilateral NAcc (Fig. 4*B*, F(1,48) = 4.302, *P*_uncorrected_ = 0.043), with a stronger variance negative modulation on BOLD signals for the broader option, an effect not seen for vmPFC (*P*_uncorrected_ = 0.250).

## Discussion

Combining a magnitude learning task and ultra-high resolution fMRI, we investigated mechanisms underlying the emergence and expression of individual differences in provariance bias. We show that individual differences in learning rate bias, derived from a 2lr-RW model, are associated with provariance biases, which in turn are related to task-independent individual differences in anxiety and depression scores. Neurally, these individual differences in learning rate bias are associated with the strength of functional connectivity between habenula (Hb), a region implicated in encoding NPEs, and VTA in response to NPEs.

We first identified regionally distinct responses to model-derived PPEs and NPEs in dopaminergic regions including NAcc, VTA, and vmPFC, areas previously implicated in reward prediction error encoding in humans (see ref. 48 for a meta-analysis). However, few studies have previously examined the joint expression of positive and negative PEs specifically. Notably, we show NAcc BOLD responses were positively modulated by PPEs and negatively by NPEs, a finding consistent with previous fMRI evidence showing ventral striatum sensitivity, including NAcc, to both unexpected reward and reward omission (49, 50). On the other hand, VTA was sensitive to PPEs but not NPEs, in line with previous human fMRI studies showing VTA expresses significant BOLD responses to unexpected rewards but not reward omission (50), and a PPE-like signal in an electric shock expectation task (50, 51). By contrast, we found vmPFC was significantly modulated by NPEs alone, consistent with other human-fMRI evidence that vmPFC BOLD responses were selective for unexpected reward omission (52) as well as the expression of an NPE signal in a fear conditioning task (53).

Importantly, leveraging ultra-high-resolution fMRI, we show that human Hb encodes trial-by-trial NPEs, consistent with animal evidence that showed lateral habenula plays a key role in value-guided behavior (for a review see ref. 54). Heretofore, its small size and relatively low resolution of 3T MRI have meant its role in human value-based learning has remained elusive. Studies using 3T fMRI report heightened BOLD responses in Hb to aversive outcomes, such as primitive negative events (shock) (38, 39, 55, 56), negative feedback (57), punishment (58), a high probability of loss (59), and cued primary reward (juice) omission (60). However, studies have not shown Hb BOLD responses are modulated by monetary loss (38, 39), though a recent 7T fMRI study showed relative BOLD Hb deactivation to monetary loss avoidance (61). Here, by combining ultra-high-resolution imaging and computational modeling, we go a step further and provide human evidence that Hb encodes trial-by-trial NPEs in a monetary reward task.

In our magnitude learning task, a negative bias in learning rates, as derived from a 2lr-RW model, captured individual differences in a provariance bias. This finding coupled with the observation that a more negative Hb-VTA functional connectivity during NPEs was associated with an enhanced negative learning rate bias, highlights the importance of this pathway in risk-aversive behavior. Thus, we suggest that Hb-VTA modulation of a learning rate bias may contribute to the emergence of individual differences in risky behavior. Evidence consistent with this mechanistic account includes evidence from rodents that stimulating a Hb-VTA pathway induces conditioned place aversion (62). Note that the resolution of human noninvasive neuroimaging does not enable examination of the lateral habenula specifically, and our region of interest includes both lateral and medial habenula. Lateral habenula alone is linked to expression of NPEs, while medial habenula has a different functional attribution (63–65). However, despite this limitation in resolution, we provide evidence that human habenula encodes NPEs, and through its functional connectivity to VTA exerts an influence on learning rate biases. Although we showed that VTA BOLD responses were not modulated by NPEs directly, our results suggested VTA might be involved in the network processing NPEs. These results highlight the functional interactions between reward regions in modulating behavior rather than local BOLD activities, which represent overlapping but separate neurophysiological processes from functional interactions (66, 67). However, we caution that this could also be due to limited fMRI sensitivity to more subtle neural modulations, as VTA exhibits stronger excitation to PPEs than its suppression by NPEs suggested by animal evidence (22).

An enhanced negative BOLD response in NAcc to NPEs was associated with a more negative learning rate bias; however, the effect was not strong in our study. But, consistent with our results, we note a previous fMRI study showed that a differential NAcc BOLD response to prediction errors for risky options, relative to certain options, is linked to behavior risk preferences (68). In the aversive domain, a previous finding from our lab also showed a negative modulation of striatal responses by aversive prediction errors (delivery of shock) linked to a more negative learning bias and a reduced propensity to gamble (69). Therefore, we further investigated this with a mediation analysis, which suggests that Hb-VTA coupling impacts NAcc responsivity to NPEs via an influence on learning rate biases (mediator). We speculate that the association between a striatal involvement and a learning bias, as seen both here and in an earlier study (69), may reflect a modulation by upstream reward regions, specifically a functional interaction between Hb and VTA.

Intriguingly, we found evidence for adaptive coding of reward prediction errors to outcomes in NAcc and vmPFC. Specifically, in both regions, item-based reward prediction error signals were enhanced under higher variance, consistent with an uncertainty boosting of RPE signals. Consistent with our results, uncertainty has been shown to increase an outcome signal in NAcc (70). In nonhuman primates, both reward risk (variance) and reward volatility (unexpected uncertainty) increase neural activity in the orbital frontal cortex (OFC) (71, 72), a region with functional attributions similar to vmPFC in humans (73). However, other evidence, using an instructed cue learning task whereby participants were informed regarding the SD of outcomes, showed this served to reduce RPE signals in the ventral striatum (74). We suggest that, as in our task, when subjects are actively learning variances then this serves to boost prediction error signals, as opposed to when variances are well learned or instructed, which results in a consequential decrease in prediction error signals. Indeed, a study compared learned and described risk during a risk decision-making task showed that the expected value signal is higher in vmPFC for the learned risky option (75). Consistent with this is evidence for a positive encoding of uncertainty during exploration (in learning) and negative encoding during exploitation (well learned) in the vmPFC (76). Interestingly, we showed that the RPE for the unchosen option is also modulated by variance in NAcc, however, this effect is not seen for vmPFC. However, we caution that because of the limit of our task design (limited trial number per block), we could not examine the effect of variance on PPEs and NPEs separately but instead combine PPEs and NPEs and examine prediction errors in general. This could also explain why we found a stronger variance effect on reward prediction error signal in NAcc, which was significantly modulated by both PPEs and NPEs, but not other ROIs.

A reduced provariance bias, and a negative bias in learning rates, were associated with higher anxiety and depression scores. Negative cognitive bias has been a core cognitive model of depression (77). We show that negative learning bias is modulated by Hb-VTA functional coupling to NPEs and this aligns with evidence indicating Hb plays a major role in depression (78). For example, an animal depression model (cLH rats) has shown increased spontaneous lateral habenula activity (bursting), which can be attenuated by ketamine, a fast-acting antidepressant (79). Notably, in macaque monkeys, ketamine has been shown to increase provariance bias (11) while in rodents inactivation of Hb leads to more positive bias learning accompanied by reduced deactivities to reward omission in DA neurons in VTA (26). Together, these results showed a link between the habenula–dopamine pathway to negative bias in depression.

There are other limits in this study that we address here. First, we showed an intriguing finding that provariance bias was enhanced when both options had means higher than the global means (i.e., 7 for a poker distribution). This finding has been replicated in several of our online studies using the same paradigm (see ref. 8 for details), as well as a study using a different task (80). We developed a Bayesian-CvaR model that captures both this effect and individual differences in PVB in the previous computational work (8). However, the Bayesian-CVaR model was not the best-fitted model in this fMRI study. Therefore, we analyzed our data based on the winning model in this current study, i.e., the 2lr-RW model. However, we show the main results were replicated with estimates from a Bayesian-CVaR model (*SI Appendix*, Fig. S12). Second, the main motivation of this study was to investigate neural mechanisms underlying individual differences in risk preference, i.e., PVB. We aimed to repeat as many equal-mean blocks given the time constraint of an fMRI study, at the expense of trial number within a block. This limited us to an investigation of within-block learning dynamics. Although we focus on the question of irreducible uncertainty in the current study (i.e., the variances in the distributions), it would be of interest in future studies to examine how estimates of the mean and variances evolve over trials (reducible uncertainty). For this purpose, one might consider adding more explicit tests of knowledge of variances, which were also lacking in the current study. Finally, we used a study-specific Hb ROI, which is mostly applied for Hb fMRI studies (37–39, 81), because for such a small region the misalignment might be quite significant with an independent Hb ROI mask (*SI Appendix*, Fig. S14). However, this is a limit that one should be mindful of in such a study.

In conclusion, we show that a negative bias in learning rates, which accounts for individual differences in provariance bias, relates to an enhanced Hb-VTA functional coupling for NPEs. By modulating learning rate bias, the Hb-VTA functional interaction influences downstream region NAcc’s response to NPEs. The results link learning signals in the brain and PVB and provide a neural basis for individual differences in risk preference, with implications for psychopathology.

## Methods

### Participants and Procedures.

In an fMRI study, we recruited 57 participants from the general population. To cover a spectrum of anxiety and depression levels, participants were prescreened on their scores on anxiety and depression questionnaire scores, measured by the Zung SDS and the STAI. We adopted the same behavior data control criteria as our previous study (8). More specifically, five subjects were excluded from the analyses because their mean accuracies for the different-means blocks were less than 60%; one was excluded because they chose the same option for all the trials in at least one of the four same-mean blocks; a final subject was excluded for both reasons. One additional participant was excluded on the basis that they showed a provariance bias that exceeded 3 SD of the overall group mean. This resulted in a final sample of 49 (34 females) participants, aged 18 to 30.

The experiment was implemented using the software PsychoPy (v2021.1.4) (82). Each participant completed 8 blocks of the learning task in a 7T MRI scanner, with a rest between blocks. Participants were reimbursed a base amount plus a bonus earned during the learning task. All participants completed 5 questionnaires on the scan day measuring different cognitive traits associated with anxiety and depression (83): 1) the STAI (84); 2) the Zung SDS (85); 3) the rumination response scale (RRS) (86); 4) the intolerance of uncertainty scale (IUS) (87); 5) the 17-item Dysfunctional Attitude Scale form A (DAS-A) (88). We used their questionnaire scores taken on the scanning day for all the relevant analyses.

All participants provided informed consent before any testing. This study was approved by the Beijing Normal University Research Ethics Committee (IRB_A_0051_2021001) in accordance with the Declaration of Helsinki (89).

### The Experiment Task.

We adapted a magnitude learning task, as reported in a previous paper (8), wherein participants made choices between two card decks with varying hidden card number distributions for 30 trials in a block (Fig. 1*B*). For each block, the same two card decks, cued by different colors and/or patterns of the back of the poker cards, were shown throughout. Two new card decks (different colors and/or patterns from the ones shown before) were assigned to each new block. Each participants completed eight blocks in total in the scanner: four different-mean blocks: one narrow-high vs. broad-low (NHBL) block, one broad-high vs. narrow-low (BHNL) block, and two broad-high vs. broad-low (BHBL) blocks; and four same-mean blocks: two broad-high vs. narrow-high (BHNH) and two broad-low vs. narrow-low (BLNL). Note that, compared to the previous paper, we did not include the bimodal block which did not show any correlation with individual differences in anxiety-and-depression-related traits (see ref. 8 for more details). The blocks were presented in a pseudorandom order so that 1) half of the participants started with an equal-mean block and the other half a different-mean block, 2) within a participant, there were always two of the equal-mean blocks and two of the different-mean blocks in the first half of the session, and the rest of four blocks in the second half. 3) an equal-mean block was followed by another equal-mean block or a different-mean block with the same probabilities and vice versa for a different-mean block. Participants were not informed about block types. Prior to the scanner, participants were introduced to the task and given a different-mean block as a practice block.

At the beginning of each trial, participants were presented with two pictures of the back of the card decks and asked to choose (self-paced) one of them. The positions (left or right side) of the two card decks were randomized from trial to trial. When they completed a choice, the cross in the center of the screen changed into a triangle and pointed to the card deck they had chosen for the trial. One to three (mean 2 s) seconds after a choice, the card number from one of the decks was revealed for 1 to 3 s (mean 2 s) and then turned back. Half a second later, the card number from the other card deck was revealed for 1 to 3 s (mean 2 s). The order was counterbalanced across trials such that half of the time one card deck was revealed first. Finally, the card numbers were shown together again for 1 s. Simultaneously, participants were shown how many points they had won or lost for that trial. If the chosen card number were higher than the unchosen card number, participants gained points equal to the value difference between the two numbers for that trial; if the chosen card number was lower than the unchosen one, participants lost points equal to the value differences between the two cards. If the two card numbers were equal the total points did not change for that trial. To encourage better performance, points accumulated in the magnitude learning task were converted into a monetary bonus, which was paid to participants together with a fixed amount reimbursement. Participants were informed of this before they started the task.

### Behavior Data Analysis.

#### Statistical analysis.

The percentages of choosing the higher option (for the different-mean blocks) or the broader option (for the same-mean blocks) were calculated for each block type respectively. The mean provariance biases were calculated by averaging the percentages of choosing the broader options for all four same-mean blocks, i.e., two BHNH blocks and two BLNL blocks. Using one-sample *T* tests, we tested whether participants’ percentages of choosing the higher or broader option were different than the chance level (50%) for each block type, respectively. Paired *T* tests were used to compare participants’ overall performance between different block types. Correlation analyses were performed using Pearson’s Correlation. The statistical analyses were performed in IBM SPSS Statistics, version 26.0.0.0^®^. All stats were two-tailed.

Bayesian one-sample *t* tests (JASP (Version 0.19.3) (90)) were used to quantify evidence for/against null hypotheses. BF_+0_ and BF_-0_ were used when specifically examining a positive or a negative effect, respectively. BF_10_ was used when examining whether the effect is different than zero.

#### Computational modeling.

Our winning model is a two-learning-rate Rescorla–Wagner model (2lr-RW) (91), a simple variant of the RW model (12). Please refer to our previous computational work (8) and *SI Appendix* for a detailed description of the alternative models and model comparisons. In this model, two different learning rates (α_+_ and α_−_) are assigned to the PPEs and NPEs, respectively (Eq. **1**). More specifically, for every trial, using Eq. **2**, a prediction error (δ) was calculated using the outcome received for the current trial (R_t_) minus the expected value for the current trials (V_t_). The expected value for the next trial (V_t+1_) is updated using Eq. **3** by taking the EV from the previous trial (V_t_), adding the product of prediction error times and a learning rate α(δ) dependent on the sign of the prediction error for the current trial: α_+_ is used if this prediction error is positive (i.e., higher than or equal to 0); α_-_ is used if this prediction error is negative (i.e. lower than 0) (Eq. **1**).

Bias in learning rates would lead to a bias in updating distribution variances, making the expectation (V_t_) higher or lower than the true mean of a distribution. The negative learning rate bias (LR_neg-bias_) is calculated using Eq. **4**. In previous work, we showed in simulation the learning rate bias would generate a spectrum of provariance biases in the paradigm deployed in the current study (8). The outcome values (i.e., 1 to 13) were rescaled to 0.01 to 0.99 and the prior expected value was set to 0.5.

[1] $$\alpha_{\left(\delta \right)}=\left\{\begin{array}{c} \alpha_{+},\mathrm{if}\;\delta \geq 0\\ \alpha_{-},\mathrm{if}\;\delta <0 \end{array}\right.,$$

[2] $$\delta =R_{t}-V_{t},$$

[3] $$V_{t+1}=V_{t}+\alpha_{\left(\delta \right)}\cdot \delta ,$$

[4] $${\mathrm{LR}}_{\mathrm{Neg}-\mathrm{bias}}=\frac{\alpha_{-}}{\alpha_{+}+\alpha_{-}}.$$

For a two-arm bandit choice task, the probabilities of choosing an option are linked to the difference between the learned expected values for the two options. We adopted a standard softmax function for decision-making processes to generate a probability of choosing option a (Eq. **5**) (92) where inverse temperature β controls the stochasticity of decision-making. Note that Eq. **5** is also used in other models in the current study (see *SI Appendix* for details of other models used).

[5] $$P_{a}=\frac{1}{1+e^{-\beta \cdot \left(V_{a}-V_{b}\right)}}.$$

All models were fitted in Matlab R2020b using a variational Bayes approach. Only behavioral data from the four same-mean blocks were used for the model fitting. This is because the different-mean blocks were relatively simple, and a majority of participants always chose the higher option in these blocks. All trials from the same-mean blocks were used in modeling fitting. Bayesian information criterion (BIC) was calculated for each model using the best-fitted parameters for each participant (Eqs. **6** and **7**). LÌ‚ denotes the maximized value of the likelihood function of the model M, x: the observed data, n: the number of observations, k: the number of free parameters in the model. BICs for a random model were calculated with each decision probability set as 0.5 and k = 0. BIC scores were summed across participants, with lower sum BIC indicating better model fit. Delta BIC for each model was calculated by subtracting the BIC score of the best-fitting model in each experiment.

[6] $$BIC=kln(n)-2ln\left(\hat{L}\right),$$

[7] $$\hat{L}=p(x|\hat{\theta },M).$$

We then used the best fitted parameters for each participant to calculate the trial-by-trial prediction errors which were then used in GLM 1 for the first-level fMRI data analyses.

### fMRI Data Analysis.

#### Imaging data acquisition.

Imaging data were collected using a Siemens 7T MRI scanner in Peking University in Beijing, China. Functional data were acquired using a multiband gradient-echo T2* echo planar imaging (EPI) sequence with a 1.2×1.2×1.2 mm resolution; multiband acceleration factor 2; repetition time (TR) 1,375 ms; echo time (TE) 19 ms; flip angle 60°; and a GRAPPA acceleration factor 3. Field of view (FOV) was adjusted to cover the basal ganglia and thalamus; see *SI Appendix*, Fig. S3. During the main functional runs, cardiac and respiratory frequencies were collected to regress out the effect of physiological noise. Additionally, a single whole-brain functional image was acquired after the main functional runs with the same parameters except for a TR of 3,298 ms.

Structural data were acquired with a T1-weighted MP-RAGE sequence with a 0.7×0.7×0.7 mm resolution; GRAPPA acceleration factor 2; TR 2,200 ms; TE 1.65 ms; and inversion time (TI) 1,050 ms. A separate Fieldmap sequence was acquired with 2×2×2 mm resolution; TR 620 ms; TE1 4.08 ms; TE2 5.10 ms.

#### fMRI data preprocessing.

Preprocessing was performed using tools from FMRIB Software Library (FSL) (93). Functional images were first normalized, spatially smoothed [Gaussian kernel with 2 mm full-width half-maximum (FWHM)], and temporally high-pass filtered (3 dB cut-off of 90 s). Participants’ head motion during the scanning was removed by MCFLIRT (94). The Brain Extraction Tool (BET) (95) was used on functional and structural images to separate brain from non-Brian matter. Structural images were bias field corrected before BET. The functional images were registered to Montreal Neurological Institute (MNI)-space in three stages: 1) partial FOV task EPI to whole-brain EPI using FMRIB’s Linear Image Registration Tool (96). 2) Whole-brain EPI to individual structural image using Boundary-Based Registration (BBR) (97) by incorporating Fieldmap correction. 3) Individual structural image to Standard image by using FMRIB’s Non-Linear Image Registration Tool (FNIRT).

#### Model-based fMRI data analyses.

Statistical analyses of the fMRI data were performed at three-levels using FSL FEAT (98). At the first level, univariate general linear models (GLMs) were run for each session/block for each participant.

##### GLM 1.

BOLD = β_0_+ β_1_ × Response at choice phase + β_2_ × reaction time at choice phase+ β_3_ × ones at choice phases (capturing the mean of the choice phase) + β_4_ × PPEs at the chosen option reveal (1st or 2nd feedback) + β_5_ × ones (mean) at the chosen option reveal with PPEs + β_6_ × NPEs at the chosen option reveal (1st or 2nd feedback) + β_7_ × ones (mean) at the chosen option reveal with NPEs + β_8_ × PPEs for the unchosen option reveal (1st or 2nd feedback) + β_9_ × ones (mean) at the unchosen option reveal with PPEs + β_10_ × NPEs at the unchosen option reveal (1st or 2nd feedback) + β_11_ × ones (mean) at the unchosen option reveal with NPEs + β_12_ × ones (mean) at the 3rd feedback. (Note that NPEs were flipped in signs so that the higher the number the more the outcome negatively deviated from the expected value.)

##### GLM 2.

BOLD = β_0_ + β_1_ × Response at choice phase + β_2_ × expected value difference between the two options at choice phase + β_3_ × ones (mean) at the choice phase + β_4_ × prediction errors for the broader option when it was chosen and its outcome revealed + β_5_ × ones (mean) for the broader option when it was chosen and its outcome revealed + β_6_ × prediction errors for the broader option when it was not chosen and when its outcome revealed + β_7_ × ones (mean) for the broader option when it was not chosen and when its outcome revealed + β_8_ × prediction errors for the narrower option when it was chosen and when its outcome revealed + β_9_ × ones (mean) for the narrower option when it was chosen and when its outcome revealed + β_10_ × prediction errors for the narrower option when it was not chosen and when its outcome revealed + β_11_ × ones (mean) for the narrower option when it was not chosen and when its outcome revealed + β_12_ × ones (mean) at the 3rd feedback.

The choice phase refers to when the two options were presented to participants to choose at the beginning of each trial. First feedback refers to when the outcome of the first card was revealed to participants in a given trial. Second feedback refers to when the outcome of the second card was revealed to participants in a given trial. Third feedback refers to when the outcome of both cards was shown simultaneously to participants in a given trial. See Fig. 1*A*.

For GLM 1, at the second level, the contrast of parameter estimates from all sessions/blocks was combined for each participant using fixed effects. For GLM 2, at the second level, the contrast of parameter estimates from the same block type (i.e., the two BHNH blocks and two BLNL blocks) was combined for each participant using fixed effects.

The subject mean contrasts from the second level were put into a mixed effects model (FLAME1) (99) for the group-level analyses.

### PPI analysis.

In order to examine task-related interregional interactions among brain regions, that were modulated by PPEs and NPEs, we implemented PPI analyses using FSL (100) and where bilateral Hb ROI was the seed region. Based on *GLM 1*, we ran PPI for the NPEs and PPEs parametric regressors, respectively.

### ROIs.

The habenula ROIs were manually drawn on each participant’s high-resolution structural scans according to an established protocol (37). The Hb ROIs in individual spaces were then transferred to standard MNI space and combined to create study-specific Hb ROIs for left, right, and bilateral Hb ROI masks respectively. The Hb ROIs in the MNI space were thresholded such that the voxels were shared by more than 30% of the participants (i.e., 15). Additionally, we used anatomical masks (28) for NAcc and VTA, and a functionally defined ROI for the vmPFC based on a meta-analysis of value-based decision-making tasks (Fig. 2 *B*–*D*) (36).

ROI analyses for PPEs, NPES, and PPI analyses were performed by extracting beta estimates from the whole-FOV contrast images using the fslmeants function with each ROI mask.

### SEM.

SEM enables assessment of hypothetical causal relations among several (more than two) continuous variables based on their covariance with one another (43, 45). All SEM was conducted in Latent Variable Analysis (lavaan) package v.0.6-17 using Maximum Likelihood estimation (44).

Based on the interaction of the ROIs (Hb, VTA, NAcc, and mPFC) shown in animal studies (46), we constructed the hypothesized relationships among the 4 ROIs as shown in Fig. 3*D*, i.e., Hb to VTA and then VTA to NAcc and mPFC (Hb→VTA→NAcc/vmPFC). We then permute ROI positions within this structure (i.e., A→B→C/D), which results in 12 combinations (*SI Appendix*, Fig. S8*b*). Matrixes comprising the filtered time series of BOLD signals in each ROI from all blocks were used. AIC was used for model comparison. The winning model was the one with the lowest AICs of all 16 combinations. Delta AIC for each model was calculated by subtracting the AIC score of the winning model for each combination (In *SI Appendix*, Fig. S8*A*, delta AICs were plotted with x labels corresponding to the starting ROI in this structure).

### Mediation Analysis.

A mediation analysis using the multilevel mediation and moderation (M3) toolbox (47) in Matlab with permutations (n = 50,000) was performed to examine the relationships among Hb-VTA functional connectivity, NAcc BOLD responses to NPE and a negative bias in learning rates. In the analysis, negative biases in learning rates were set as the mediation factor, and Hb-VTA PPI connectivity to NPEs and NAcc BOLD modulation parameter estimates by NPEs as independent and dependent variables, respectively.

### Statistics and Multiple Comparisons for ROI Analyses.

Two-tailed one-sample *T* tests were used to test modulation effect of PPEs and NPEs (against 0), respectively, for each ROI using IBM SPSS, version 26.0.0.0^®^. Adjusted *P* values were calculated using the Python package “statsmodels” (0.14.4) with the Benjamini–Hochberg Procedure for Controlling the False Positive Rate (reported as *P*_FDR_).

## Supplementary Material

Appendix 01 (PDF)

## Data Availability

Anonymized behavioral and fMRI ROI data are available at the project’s OSF directory (https://osf.io/2kcxn/) (101).
