# Supplementary material for: Habenula–ventral tegmental area functional coupling and risk aversion in humans
Source: Proc Natl Acad Sci U S A. 2025 Oct 30;122(44):e2500815122. doi: 10.1073/pnas.2500815122 (PMC12595472; doi:10.1073/pnas.2500815122)
Supplement: Supplementary file 1 — Appendix 01 (PDF) [file pnas.2500815122.sapp.pdf]

# Supporting Information for
Habenula-ventral tegmental area functional coupling and risk-aversion in humans

Wanjun Lin[12*], Jiahua Xu[3], Xiaoying Zhang[4], Raymond J Dolan[145]

1 Max Planck University College London Centre for Computational Psychiatry and Ageing Research, University College London, Queen Square Institute of Neurology, London, UK

2 Center for Functionally Integrative Neuroscience, Aarhus University, Aarhus, Denmark

3 Psychiatry Research Center, Beijing Huilongguan Hospital, Peking University Huilonguan Clinical Medical School, Beijing, China.

4 State Key Laboratory of Cognitive Neuroscience and Learning, IDG/McGovern Institute for Brain Research, Beijing Normal University, Beijing, China

5 Wellcome Centre for Human Neuroimaging, University College London, Queen Square Institute of Neurology, London, UK

**This PDF file includes:**

Supporting text
Figures S1 to S15
Tables S1
SI References

**Model free fMRI results show similar results to that of model-based fMRI analyses**

In this magnitude learning task design, participants win or lose a different number of points from trial to trial, enabling us to investigate parameter modulation of outcomes without using computational model estimates. Therefore, we examined the BOLD responses to win amount and loss amount (GLM3, see supplementary methods). As expected, BOLD responses in the NAcc (Fig.S 9) increased as participants won more points in a trial (i.e. win amount, $t(48)=6.018$ $p<.001$) and de-activated as a function of losing more points (i.e. loss amount, $t(48)=-4.405$, $p<.001$). Similar to model-based fMRI results, the habenula showed marginally significant positive modulation by loss amount in bilateral habenula ($t(48)=1.849$, $p=.071$, left habenula (Fig.S 9): $t(48)=2.158$, $p=.036$, right habenula: $p=.231$). However, mPFC (Fig.S 9) showed significantly positive modulation by win amount ($t(48)=2.724$, $p=.009$) and negative modulation by loss amount ($t(48)=-2.106$, $p=.041$). The whole-FOV analysis showed similar results to that of the ROI analysis (see Fig.S 10).

**Direct comparisons between BOLD responses to positive (PPE) and negative (NPE) reward prediction errors in each ROI**

In addition to the results presented in Fig. 2, we also ran separate paired t-tests for each ROI for PPE vs. NPE BOLD responses. This revealed a significant direct difference between PPE and NPE for NAcc ($t(48)=6.759$, $p>.001$) and mPFC ($t(48)=3.861$, $p>.001$). For VTA, we hypothesized a positive modulation by PPE, so we tested for PPE>NPE ($t(48)=1.483$, $p=.072$(one-sided)), and vice versa for Habenula (PPE< NPE, $t(48)=-1.330$, $p=.095$(one-sided)).

**An explorative outcome for variance effect on positive (PPE) and negative (NPE) reward prediction errors**

In order to see whether variance has a similar or different effect on PPE and NPE, we conducted an additional analysis (see GLM2_2 in supplemental methods), separating chosen PPE and NPE (unchosen RPEs remained combined due to low trial counts), which ended up with 15 remaining participants (so the results are underpowered and explorative). The results (see Fig.S15a) suggested variances seem to have a similar amplification effect on PPE and NPE. For example, in NAcc, in NPE, we show a stronger deactivation as a function of NPE for the broader-low option compared to narrow-low option ($t(14)=-2.055$, $p=.059$). For PPE, there was a non-significant trend toward higher activation for broader-low vs. narrow-low. Therefore, consistent with the results we showed in Fig.4, variance amplified reward prediction error modulation on BOLD signal in NAcc in general. Interesting, we also found a difference in NPE for broad-low vs. narrow low in habenula ($t(14)=-2.420$, $p=0.030$, see Fig.S15c right).

**Variances represented in the mPFC**

A recent rodent study, using neuropixels data, revelaed an abstract encoding of variance in NAcc, manifesting as a higher representational similarity between options that have similar distributions,

i.e., higher within-distribution similarity than across-distribution [1]. Using representational similarity analysis (RSA) [2] we tested for such distributional encoding in our human fMRI data. To regress out an effect of variance in response to prediction errors (parametric modulation regressors, shown in Fig 4 b&e), we ran RSA on the contrasts for the mean regressors for the chosen option for each distribution type, for the same-mean blocks and for each ROI respectively. Within both NAcc and vmPFC ROIs, we found evidence of increased representational similarity between broad-high (BH) and narrow-high (NH), as well as between broad-low (BL) and narrow-low (NL) (all $t(48)>12.89$, $p<.0001$). Additionally, in mPFC we found a significantly greater similarity between both the broader options (broad-high (BH) and broad-low (BL)) (see Fig.S 13 d, $t(48)=2.900$, Bonferroni adjusted $p=.035$). This pattern of findings is consistent with a suggestion that mPFC encodes a distribution of option outcomes, as suggested by distributional reinforcement learning theory [3].

## Supplementary Discussion

In this fMRI sample, we show that the best fitting model was the 2lr-RW model. In a previous paper [1], and in a pilot online study in China, we found that the Bayesian-CVaR model was the best fitting model. We speculate that this might reflect with the omission of bimodal blocks in the fMRI study or the relatively reduced participants size in the fMRI study, compared to the aforementioned studies. Nevertheless, we show in Fig.S 12 that all the main results for the learning rate bias estimates from the 2lr-RW model also hold true for the CVaR eta estimates from the Bayesian-CVaR model, but the effect is weaker.

## Supplementary Methods

### Statistical analysis

We ran a mixed-effects GLM model to probe effects seen using one-sample t-tests, with opt1_higher_mean encoding whether option 1 has the higher mean compared to option 2 and opt1_higher_variance encoding whether option 1 has a higher variance compared to option 2.

opt1_choice ~ 1 + opt1_higher_mean + opt1_higher_mean:opt1_higher_variance + opt1_higher_mean:opt1_higher_variance: anxiety-depression scores + (1 | subjectID).

### Online pilot study

Before conducting the fMRI study, we ran an online pilot study in China using a Chinese online study platform https://www.naodao.com/. The online pilot study used the same experimental design as that described in a previous paper [1], where a component of this pilot study was included in the supplementary [1]. The experiment was implemented using the software PsychoPy (v2021.1.4) [2]. In total, 320 participants were recruited for the online study. 107

participants were excluded based on the same pre-set criteria used here and in the previous paper [1].

**Other computational models**

**1lr-RW.** In this model, there is only one free parameter during the learning process, i.e., α, which controls how much prediction errors (δ) update the expected values (see Equation 1). Prediction errors are the differences between the outcome and the expected value for a given option.

$$V_{t+1} = V_t + \alpha \cdot \delta \qquad \text{Equation 1}$$

$$\delta = R_t - V_t \qquad \text{Equation 2}$$

**PEIRS.** In the PEIRS model, the expected values V and expected spread S of outcomes are learned simultaneously using Equation 3 and Equation 4, respectively. The estimated spread is combined with the overall prediction errors of the options δoption to determine the value V' (see Equation 6) used for the decision process (Equation 7). Stoption is the overall prediction of mean values of the two options offered compared to a global mean, which is 0.5 in this task (Equation 5). For example, for the both-high blocks, Stoption would be overall positive, Stoption for the both-low blocks, would be overall negative. ω in the Equation 6 controls the direction and how much the estimated spread would influence the decision-making process.

$$V_{t+1} = V_t + \alpha_Q \cdot \delta_{outcome} \qquad \text{Equation 3}$$

$$S_{t+1} = S_t + \alpha_s \cdot (|\delta_{outcome}| - S_t) \qquad \text{Equation 4}$$

$$S_{\text{options}}^t = \frac{V_a^t + V_b^t}{2} - 0.5 \qquad \text{Equation 5}$$

$$V_t' = V_t + tanh(\omega \cdot \delta_{options}^t) \cdot S_t \qquad \text{Equation 6}$$

$$P_a = \frac{1}{1 + e^{-\beta \cdot (V_a' - V_b')}} \qquad \text{Equation 7}$$

**Bayesian-CVaR.** In this model, a probability density function θ of the value distribution for each option is learned using Bayes' rules. The posterior belief P(θt+1) of the value distribution is updated, trial by trial, by a combination of the prior belief P(θt) and probability density function of the evidence for that trial P(Rt) using Equation 8. The initial belief P(θ0) is set as a flat distribution using a Beta distribution Beta (1,1). The probability density function of the evidence is a Beta

distribution Beta (eventαt, eventβt) with a mean of Rt and the same variance (denoted as updatevar), as shown in Equation 10 & Equation 11, respectively. eventαt and eventβt can be calculated using Equation 12 & Equation 13 respectively (these two equations were derived using the solve equation function on Equation 10 and Equation 11 in MATLAB).

$$P(\theta_{t+1}) \propto P(R_t) \cdot P(\theta_t) \qquad \textbf{Equation 8}$$

$$P(R_t) = Beta(event\alpha_t, event\beta_t) \qquad \textbf{Equation 9}$$

$$R_t = \frac{event\alpha_t}{event\alpha_t + event\beta_t} \qquad \textbf{Equation 10}$$

$$updatevar = \frac{\mathrm{event}\alpha_t \cdot \mathrm{event}\ \beta_t}{(\ \mathrm{event}\alpha_t +\ \mathrm{event}\beta_t)^2 \cdot (\mathrm{event}\alpha_t + \mathrm{event}\beta_t + 1)} \qquad \textbf{Equation 11}$$

$$event\alpha_t = -R_t \cdot (R_t^2 - R_t + updatevar)./updatevar \qquad \textbf{Equation 12}$$

$$event\beta_t = (R_t - updatevar + R_t \cdot updatevar + 2 \cdot R_t^2 + R_t^3)./updatevar \qquad \textbf{Equation 13}$$

Now that we have the trial-by-trial estimated value distributions (Z), we apply CVaR level η to read out values as input to the softmax decision-making function. CVaR can be used to read out a part of either the lower [3] or the upper end [4] of a distribution. Reading out the lowest generates the lowest value, while reading out the highest end gives the highest value. Here we set the CVaR levels η from -0.95 to 0.95, with -0.95 reading out 5% lower end of a distribution and 0.95 reading out the top 5% of a distribution, while 0 reading out the mean of the whole distribution. To do this, we first calculated the cumulative distribution function (CDF) of a distribution (Z) (Equation 14) and then find the corresponding percentile of the distribution, denoted as Value at Risk (VaR), for an $\alpha$ level (Equation 15). The CVaR is derived as either the mean of the distribution below the VaR (if η ≤ 0) or the mean of the distribution higher than the VaR (if η > 0) (Equation 16). CVaRs for the two options were put into a softmax function to estimate the probability of choosing option a.

$$F(Z) = P(Z \leqslant z) \qquad \textbf{Equation 14}$$

$$VaR_{\eta}(Z) = \begin{cases} \min\{Z \mid F(Z) \geqslant 1+\eta\}, \text{ if } -1 < \eta \leqslant 0 \\ \min\{Z \mid F(Z) \geqslant \eta\}, \text{ if } 0 < \eta < 1 \end{cases}$$ **Equation 15**

$$CVaR_{\eta}(Z) = \begin{cases} E\left[Z \mid Z \leqslant VaR_{\eta}(Z)\right], \text{ if } -1 < \eta \leqslant 0 \\ E\left[Z \mid Z \geqslant VaR_{\eta}(Z)\right], \text{ if } 0 < \eta < 1 \end{cases}$$ **Equation 16**

**Model fitting**

All models were fitted in MATLAB using a variational Bayes approach. Only behavioural data from the four same-mean blocks were used for model fitting. All trials from the same-mean blocks were used in the modelling fitting. Akaike information criterion (AIC) was calculated for each model using the best-fitted parameters for each participant (Equation 17 & Equation 18). $\hat{L}$ denotes the maximized value of the likelihood function of the model M, x: the observed data, k: the number of free parameters in the model. AIC scores were summed across participants, with lower sum AIC indicating better model fit. Delta AIC for each model was calculated by subtracting the AIC score of the best-fitting model in each experiment.

$$AIC = 2 * k - 2\ln(\hat{L})$$ **Equation 17**

$$\hat{L} = p(x \mid \hat{\theta}, M)$$ **Equation 18**

**fMRI data analyses**

**GLM 2_2:** BOLD= β0+ β1*Response at choice phase + β2*expected value difference between the two options at choice phase + β3*ones (mean) at the choice phase + β4* positive prediction errors (PPE) for the broader option when it was chosen and its outcome revealed + β5* ones (mean) for the broader option when it was chosen and had a PPE and its outcome revealed + β6* negative prediction errors (PPE) for the broader option when it was chosen and its outcome revealed + β7* ones (mean) for the broader option when it was chosen and had a NPE and its outcome revealed + β8* prediction errors for the broader option when it was not chosen and when its outcome revealed + β9* ones (mean) for the broader option when it was not chosen and when its outcome revealed + β10* positive prediction errors (PPE) for the narrower option when it was chosen and its outcome revealed + β11* ones (mean) for the narrower option when it was chosen and had a PPE and its outcome revealed + β12* negative prediction errors (PPE) for the narrower option when it was chosen and its outcome revealed + β13* ones (mean) for the narrower option when it was chosen and had a NPE and its outcome revealed + β14* prediction errors for the narrower option when it was not chosen

and when its outcome revealed + β15* ones (mean) for the narrower option when it was not chosen and when its outcome revealed + β16*ones (mean) at the 3rd feedback.

**GLM 3:** BOLD=β0+ β1*Response at choice phase + β2*reaction time at choice phase+ β3*mean of choice phase + β4* mean of the first feedback + β5* win amount for the win trials at 2nd feedback + β6* mean for the win trials at 2nd feedback + β7* loss amount for the win trials at 2nd feedback + β8* mean for the loss trials at 2nd feedback + β9* mean of the draw trials + β10* mean of the 3nd feedback (overall outcome feedback).

**Representational Similarity Analysis (RSA)**

RSA was performed using an RSA toolbox adapted for FSL data [5]. For every voxel in each of the ROI (Hb, VTA, NAcc, mPFC) and for each participant, we extracted parameter estimates from the second-level images of the GLM2 fMRI results for the mean contast for the narrower/narrower option when it was chosen and when its outcome revealed, i.e. β7 and β9 in GLM2. Then for each ROI, correlations between the voxel-based betas were calculated for each participant. We then ran one-sample t-tests to see whether the correlation coefficients were significantly different from zero across all participants.

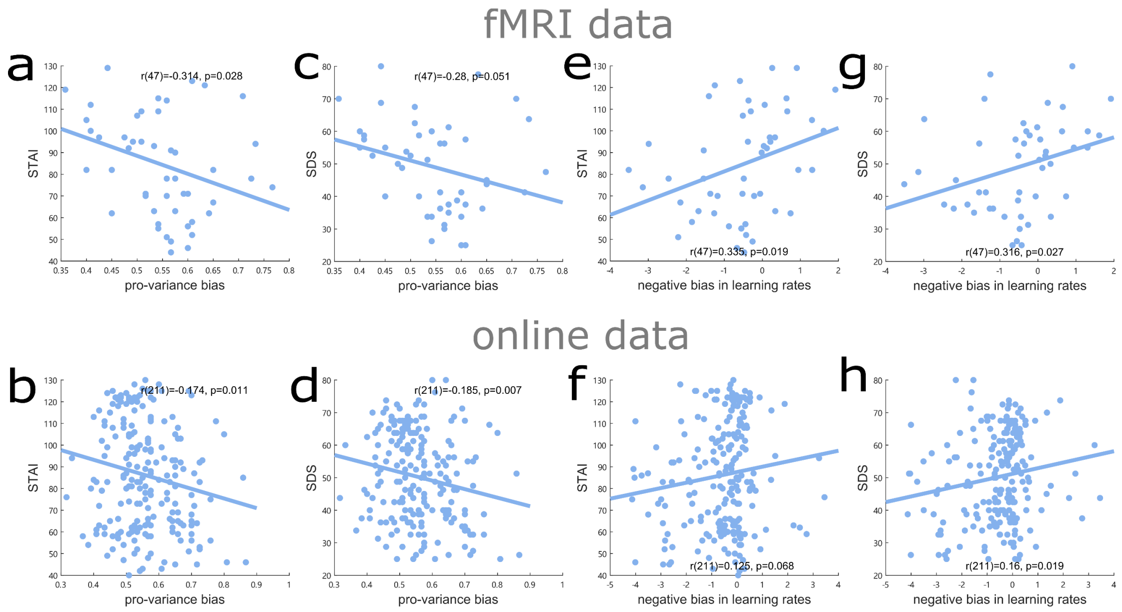


**Fig. S1. Correlations with questionnaires.** a-b) PVB negatively correlated with State-Trait Anxiety Inventory (STAI) scores for the fMRI data (a) and online data (b). c-d) PVB negatively correlated with the Zung Depression Scale (SDS) scores for the fMRI data (c) and online data (d). e-f) Negative bias in learning rates correlated with State-Trait Anxiety Inventory (STAI) scores for the fMRI data (e) and online data (f). g-h) Negative bias in learning rates correlated with the Zung Depression Scale (SDS) scores for the fMRI data (g) and online data (h).

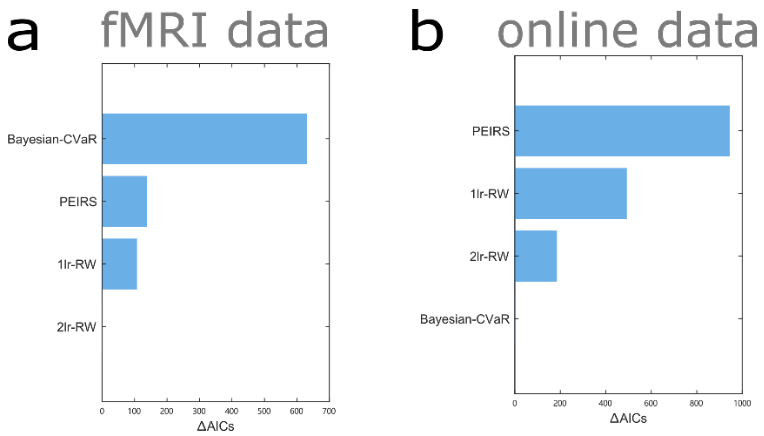


**Fig. S2. Model comparisons.** The relative Akaike information criterion (AIC) results for fMRI data (a) and online pilot data (b).

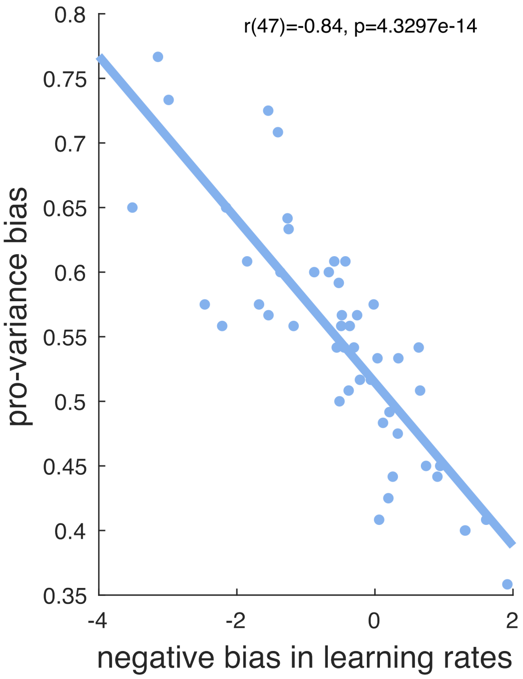


**Fig. S3. Pro-variance bias correlated negatively with negativity bias in learning rates, as derived from a 2LR-RW model.**

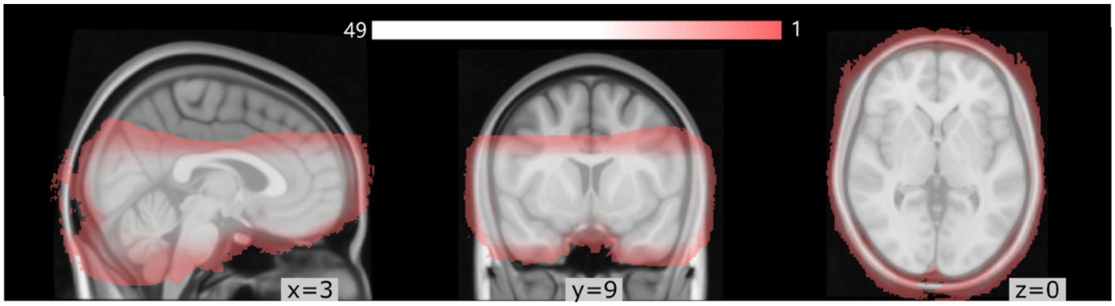


**Fig. S4. Field-of-view (FOV) for each participant after registration overlayed on a standard brain. Colormap indicates the number of participants.**

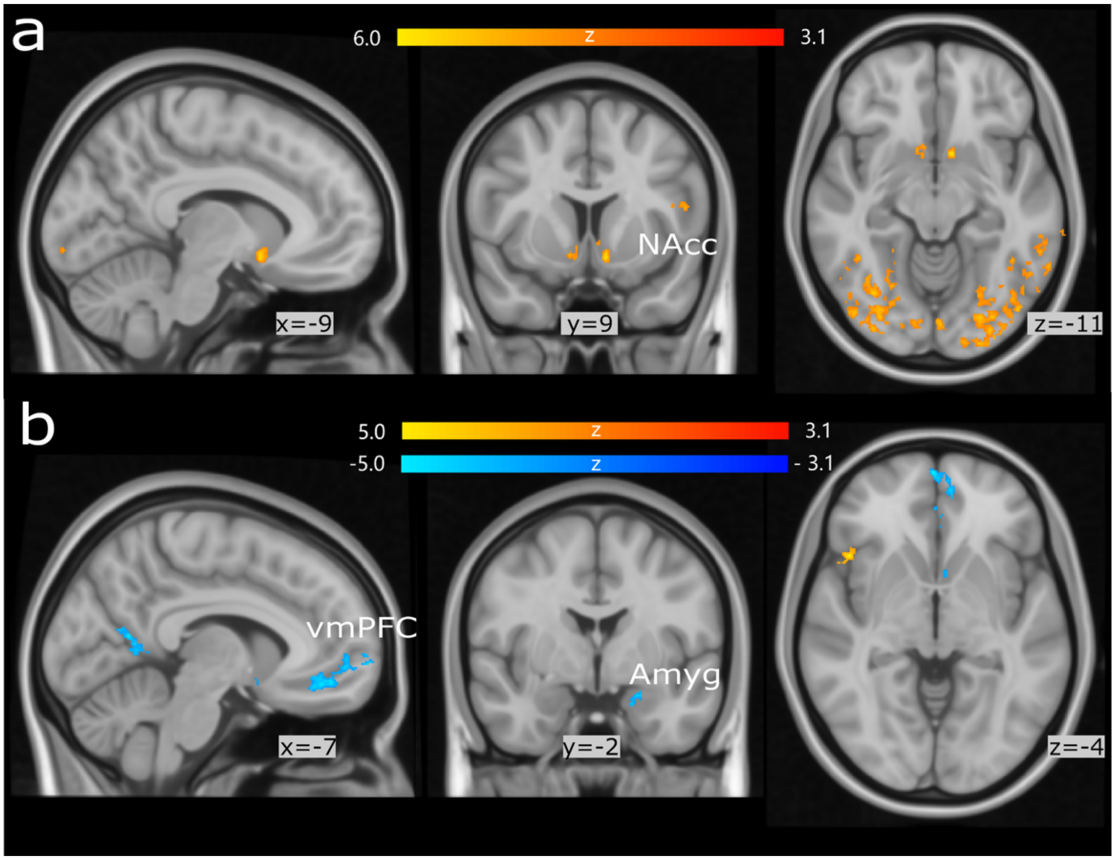


**Fig. S5. FOV results for trial-by-trial PPEs (a) and NPEs (b) parametric regressors.** Cluster $p<0.05$, $|Z|>3.1$. NAcc: nucleus accumbens; vmPFC: ventral medial prefrontal cortex; Amyg: Amygdala.

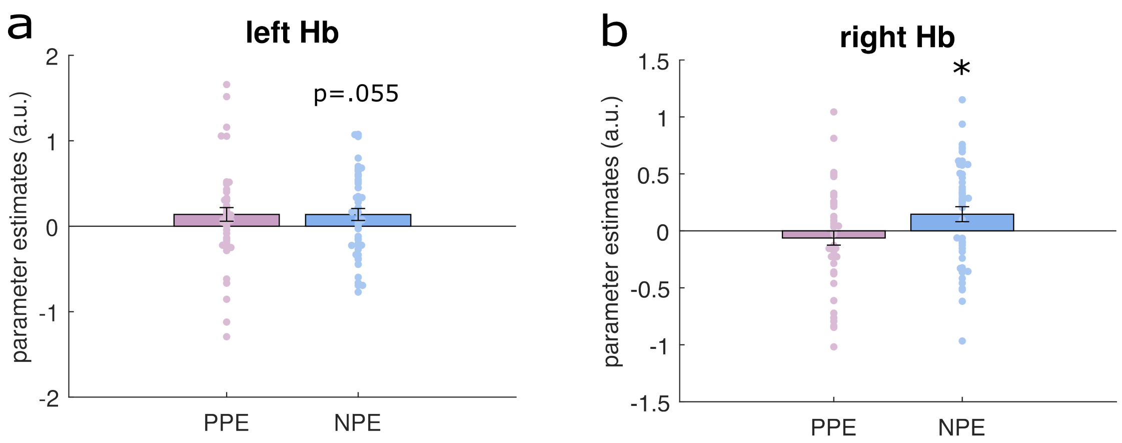


**Fig. S6. Left and right habenula (Hb) responses to PPE and NPE.** BOLD responses in both the left (a) (p= .055) and right (b) Hb (p= .031) were positively modulated by negative prediction errors (NPEs) but not positive prediction errors (PPEs). *p <.05

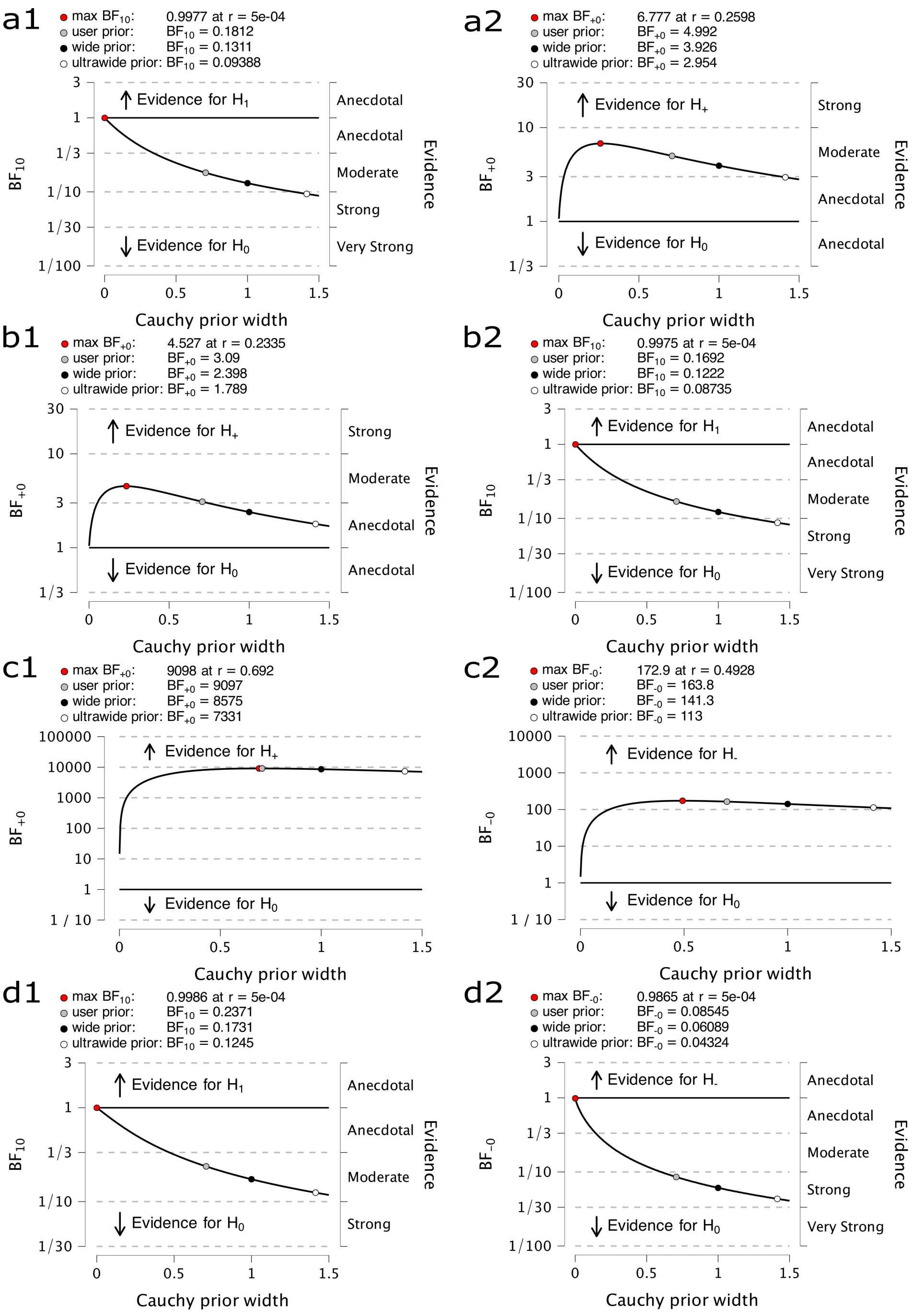


**Fig. S7. Bayes Factor Robustness check for Bayesian One-Sample t-Tests.** The plot illustrates the sensitivity of Bayes factors (BF) across a range of prior widths (Cauchy prior scale parameter *r* from 0.1 to 1.5), for 1) positive prediction error 2) negative prediction error for each ROI (a. bilateral habenula b. bilateral VTA c. bilateral NAcc and d. vmPFC) respectively. Solid lines represent BF trajectories for key comparisons. Robustness is demonstrated if $BF_{10}$ conclusions (e.g., $H_1$ support) remain consistent across plausible priors. Bayes factor at four values: the value of r that produces the maximum Bayes factor, the original user-specified prior (r = 0.707), a "wide" prior (r = 1), and an "ultrawide" prior (r = 1.414).

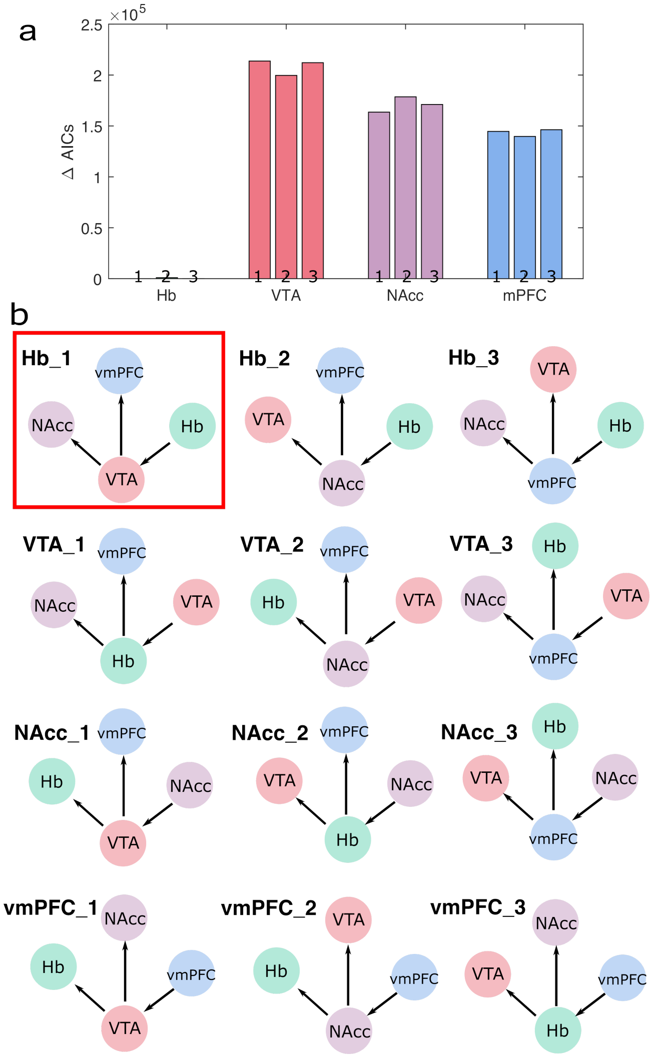


**Fig. S8. Structural Equation Modeling (SEM) results**. a) the model comparison (AICs) results for models starting from one of the four ROIs. The x-axis label denotes the starting ROI region for each model. b) the winning model (marked in red) and the alternative models for SEM analysis.

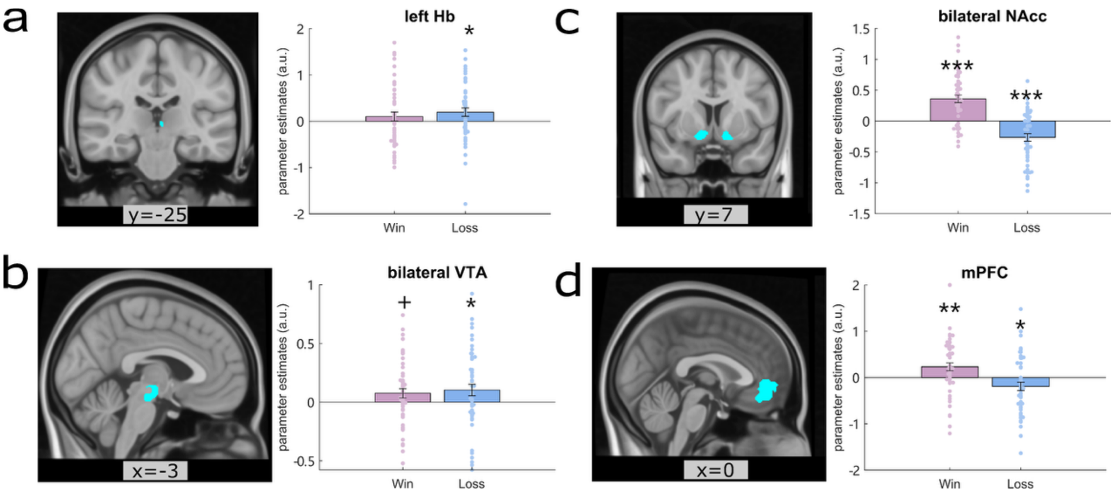


**Fig. S9. Region of interest (ROI) response to model-free outcome magnitudes.** a) BOLD responses in the left habenula (Hb) were positively modulated by loss amount (p=.036), but not by win amount (p=.785). Left: a coronal view of the left Hb anatomical mask. Right: parameter estimates of BOLD responses to win and loss amount respectively in this left Hb anatomical mask. **b)** BOLD responses in bilateral ventral tegmental area (VTA) showed a positive modulation by win amount (p=.065) and loss amount (p=.040). Left: a sagittal view of VTA anatomical mask. Right: parameter estimates of BOLD responses to win and loss amount respectively in this bilateral VTA anatomical mask. **c)** BOLD responses in bilateral nucleus accumbens (NAcc) were positively modulated by win amount and negatively modulated by loss amount (both p<.001). Left: a coronal view of the bilateral NAcc anatomical mask. Right: parameter estimates of BOLD responses to win and loss amount respectively in this bilateral NAcc anatomical mask**.** d) BOLD responses in the medial prefrontal cortex (mPFC) were positively modulated by win amount (p=.009) and negatively modulated by loss amount (p=.041). Left: a sagittal view of the mPFC functional defined mask. Right: parameter estimates of BOLD responses to win and loss amount respectively in this mPFC mask. Each dot on the bar graph represents a parameter estimate for each participant. Error bars indicate standard errors (s.e.); + p<.1 *p < 0.05; **p < 0.01; ***p < 0.001.

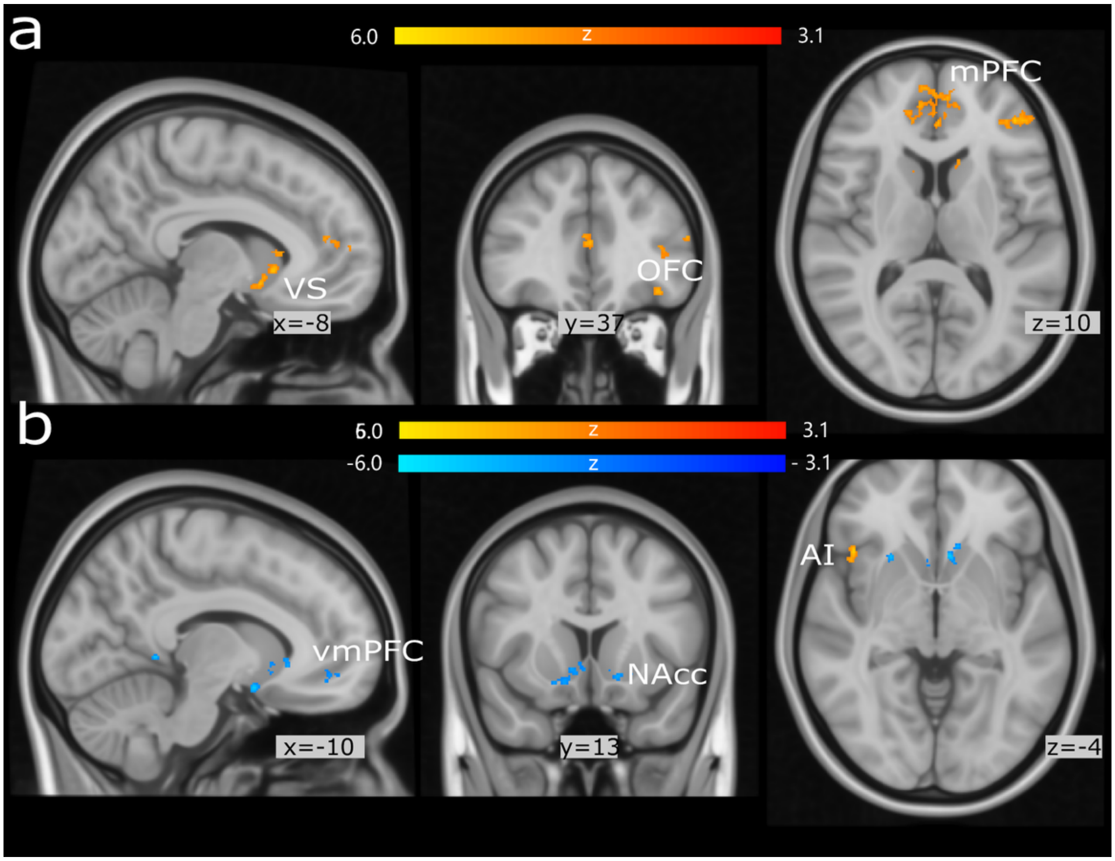


**Fig. S10. Whole brain FOV results for trial-by-trial win (a) and loss (b) amount parametric regressors.** Cluster p<0.05, |Z|>3.1. VS: ventral striatum; OFC: orbital frontal cortex; mPFC: medial prefrontal cortex; vmPFC: ventral medial prefrontal cortex; NAcc: nucleus accumbens; AI: anterior insula.

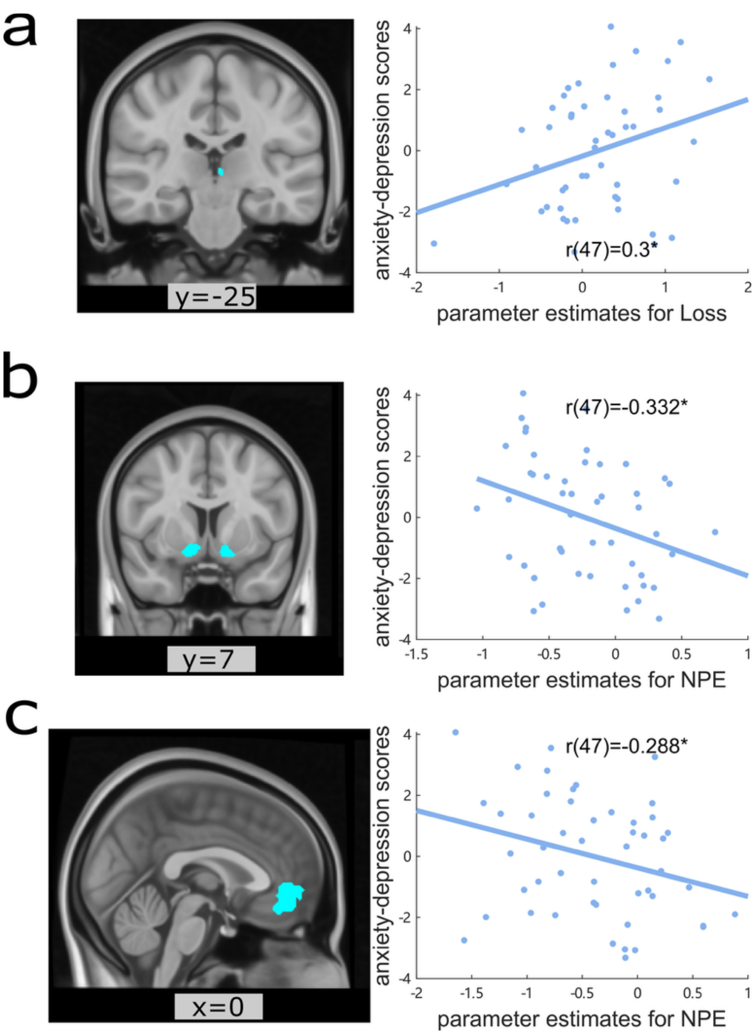


**Fig. S11. Neural correlates of negative events and anxiety-depression scores. a)** BOLD responses to loss amount in the left habenula (Hb) positively correlated with the anxiety-depression scores (p=.036). Left: a coronal view of the left Hb anatomical mask. Right: a scatter plot for parameter estimates of BOLD responses in the left Hb to loss amount (x-axis) and anxiety-depression scores (y-axis). **b)** BOLD responses to negative prediction errors (NPEs) in bilateral nucleus accumbens (NAcc) were negatively correlated with anxiety-depression scores (p=.020). Left: a coronal view of the bilateral NAcc anatomical mask. Right: a scatter plot for parameter estimates of BOLD responses in the NAcc to NPEs (x-axis) and anxiety-depression scores (y-axis). **c)** BOLD responses to negative prediction errors (NPEs) in the medial prefrontal cortex (mPFC) negatively correlated with the anxiety-depression scores (p=.045). Left: a coronal view of the mPFC functional mask. Right: a scatter plot for parameter estimates of BOLD responses in mPFC to NPEs (x-axis) and anxiety-depression scores (y-axis).

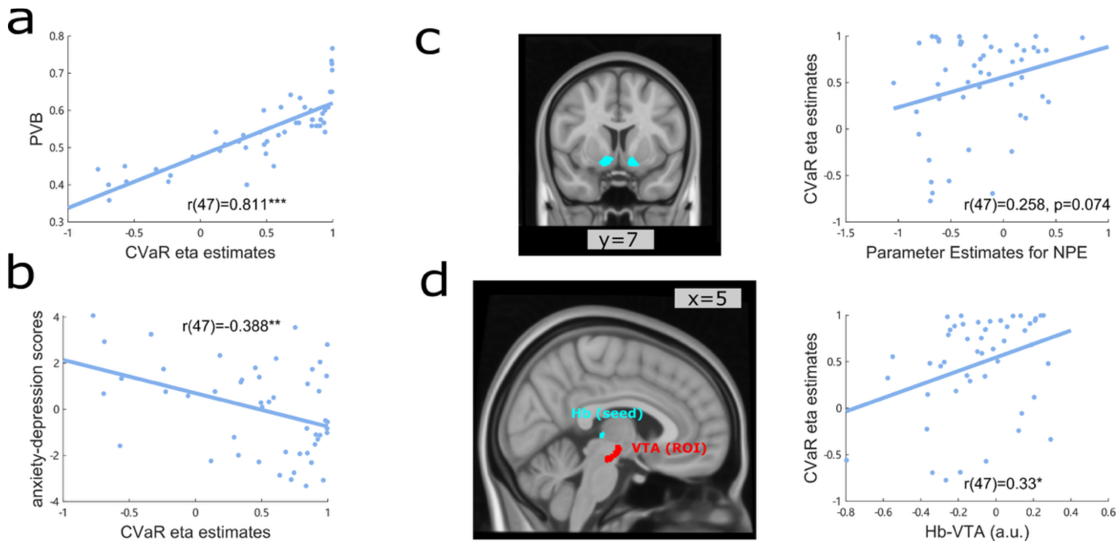


**Fig. S12. Correlation results for the eta estimated from the Bayesian-CVaR model.**

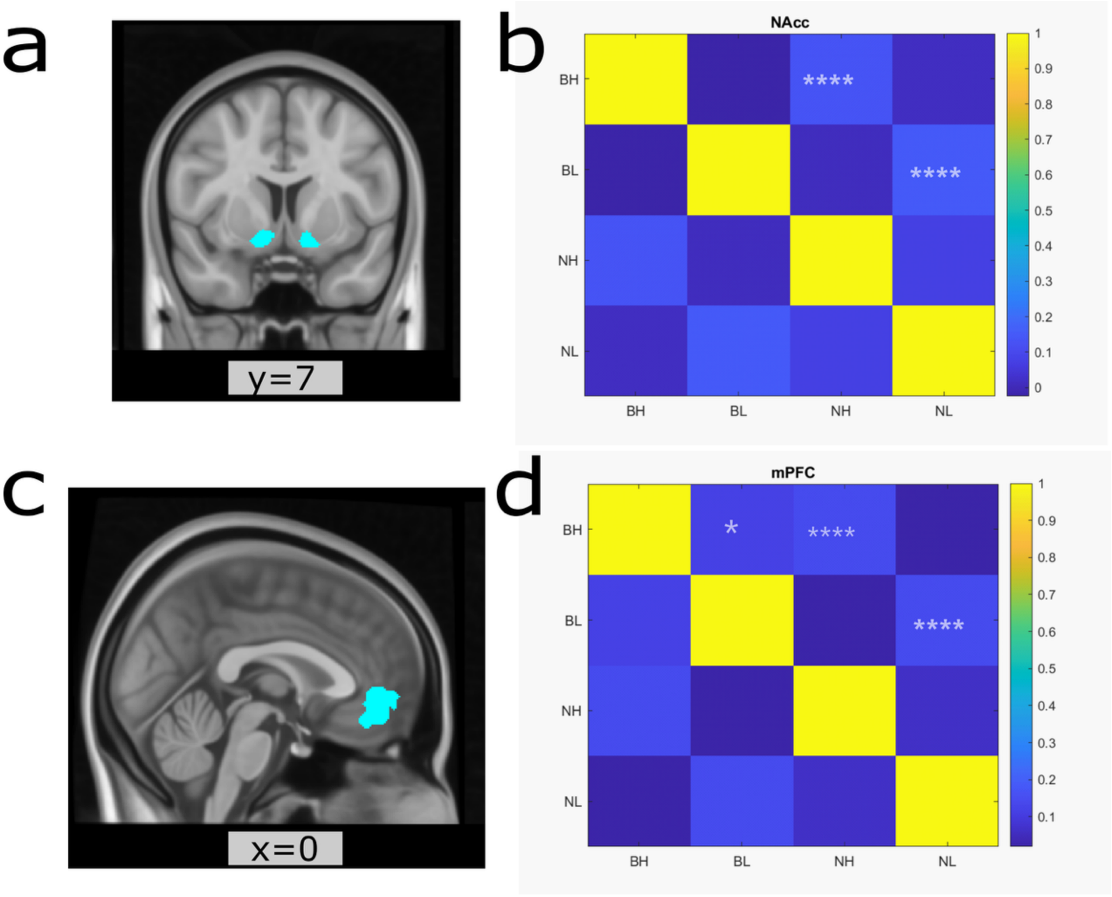


**Fig. S13. Neural representations of variances. a)** a coronal view of the bilateral NAcc anatomical mask. **b)** representation similarity in this NAcc mask between each type of option (option mean regressor) in the equal-mean blocks. **c)** a coronal view of the vmPFC mask. **d)** representation similarity in this vmPFC mask between each type of option (option mean regressor) in the equal-mean blocks. Each dot on the bar graph represents a parameter estimate for each participant. Error bars indicate standard errors (s.e.); *p < 0.05; **p < 0.01; ***p < 0.001; ****p < 0.0001.

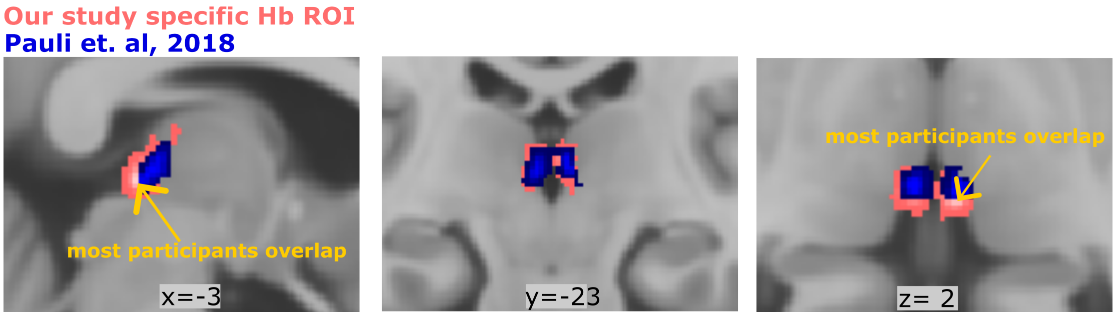


**Fig. S14. The original HB mask (in pink) is shown overlaid on mask reported by Pauli et. al (2018) mask (in blue).**

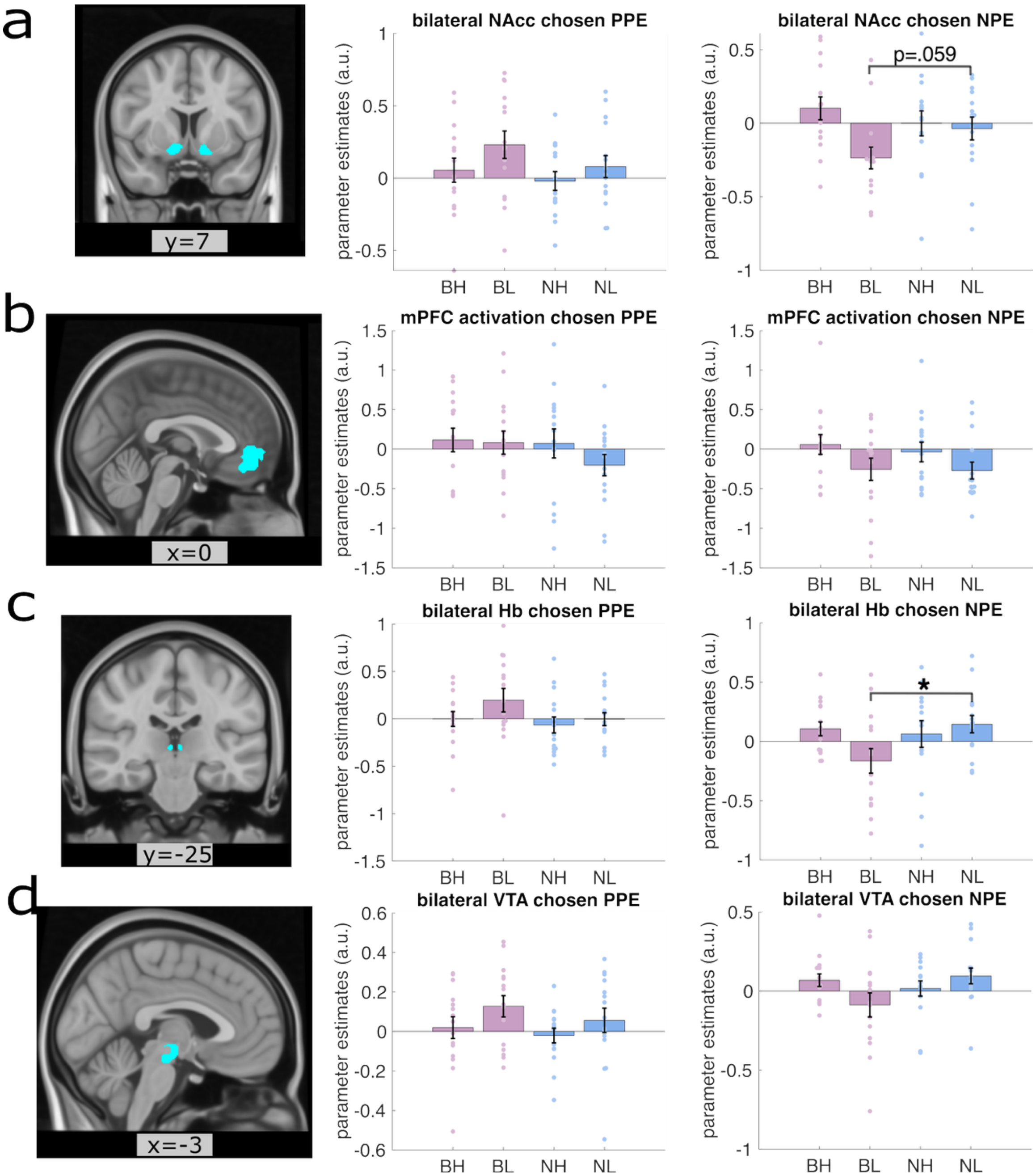


**Fig. S15. An explorative analysis to examine variance effect on chosen PPE and NPE (unchosen RPEs remained combined due to low trial counts), which 15 remaining participants with this analysis.** left column: ROI regions. middle column: parameter estimates of BOLD responses to positive prediction errors (PPE) for each distribution type respectively in the equal-mean blocks. right column: parameter estimates of BOLD responses to negative prediction errors (NPE) for each distribution type respectively in the equal-mean blocks. BH: broad-high, BL: broad-low, NH: narrow-high, NL: narrow-low. Each dot on the bar graph represents a parameter estimate for each participant. Error bars indicate standard errors (s.e.); *$p < 0.05$.

**Table S1. Estimates of the path coefficients (β) of structural equation modelling (SEM) for the winning structure** (illustrated in Fig. S8).

| **Path** | **β** | **SE** | **p** |
|---|---|---|---|
| Habenula → VTA | 0.064 | 0.001 | *<.001* |
| VTA → NAcc | 0.494 | 0.004 | *<.001* |
| VTA → vmPFC | 0.298 | 0.004 | *<.001* |

The arrows show the direction of the influence. SE is standard error. Structural equation model with statistical significance determined via two-sided Wald test as implemented in R package ‘lavaan’.

**SI References:**


1. Lowet, A.S., et al., *An opponent striatal circuit for distributional reinforcement learning.* Nature, 2025. **639**(8055): p. 717-726.
2. Nili, H., et al., *A toolbox for representational similarity analysis.* PLoS computational biology, 2014. **10**(4): p. e1003553.
3. Lowet, A.S., et al., *Distributional Reinforcement Learning in the Brain.* Trends Neurosci, 2020. **43**(12): p. 980-997.
4. Lin, W. and R.J. Dolan, *Decision-Making, Pro-variance Biases and Mood-Related Traits.* Comput Psychiatr, 2024. **8**(1): p. 142-158.
5. Peirce, J., et al., *PsychoPy2: Experiments in behavior made easy.* Behavior Research Methods, 2019. **51**(1): p. 195-203.
6. Gagne, C. and P. Dayan, *Peril, prudence and planning as risk, avoidance and worry.* Journal of Mathematical Psychology, 2022. **106**: p. 102617.
7. Rockafellar, R.T. and S. Uryasev, *Optimization of conditional value-at-risk.* Journal of risk, 2000. **2**: p. 21-42.
